# Supplementary material for: Postnatal Expression Profile of MicroRNAs Associated with Cardiovascular Diseases in 3- to 11-Year-Old Preterm-Born Children
Source: Biomedicines. 2021 Jun 24;9(7):727. doi: 10.3390/biomedicines9070727 (PMC8301298; doi:10.3390/biomedicines9070727)
Supplement: Supplementary file 1 [file biomedicines-09-00727-s001.zip › Supplementary Figure S1.pdf]

Supplementary Figure S1.

A

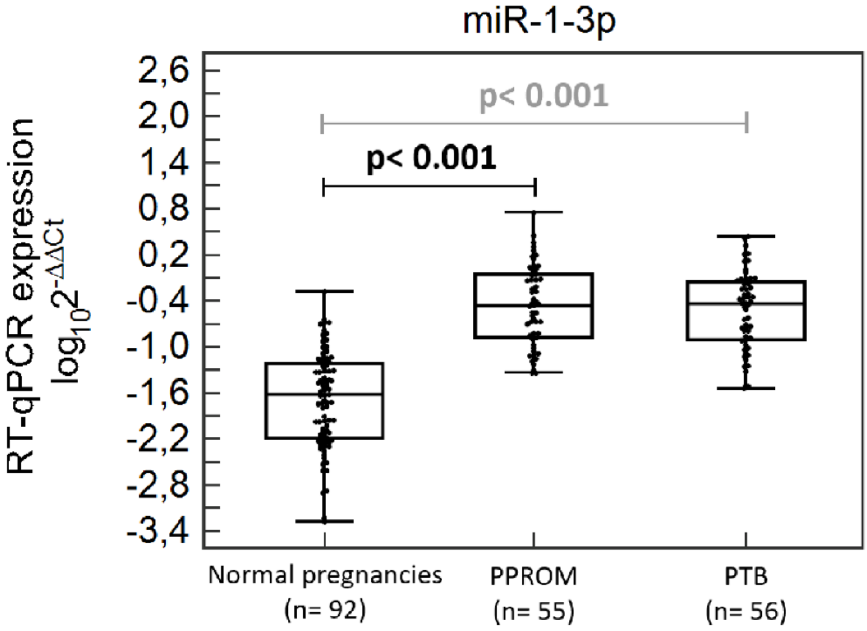

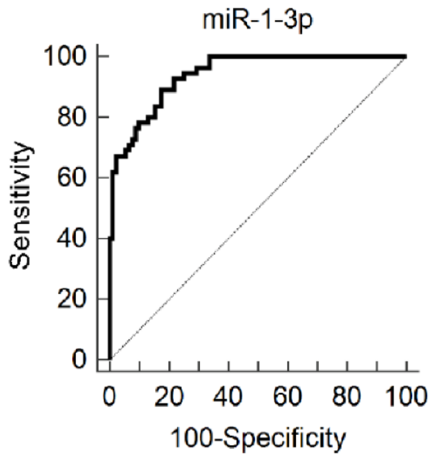

Area under the ROC curve (AUC)	0.941
Standard Error	0.0173
95% Confidence interval	0.889 to 0.973
Significance level P (Area=0.5)	<0.0001

Estimated sensitivity at fixed specificity			
Specificity	Sensitivity	95% CI	Criterion
90.00	78.18	62.23 to 90.91	>0.124151746

Criterion	Sensitivity	95% CI	Specificity	95% CI	+LR	95% CI	-LR	95% CI
>0.073354052	89.09	77.8 - 95.9	82.61	73.3 - 89.7	5.12	3.3 - 8.1	0.13	0.06 - 0.3

B

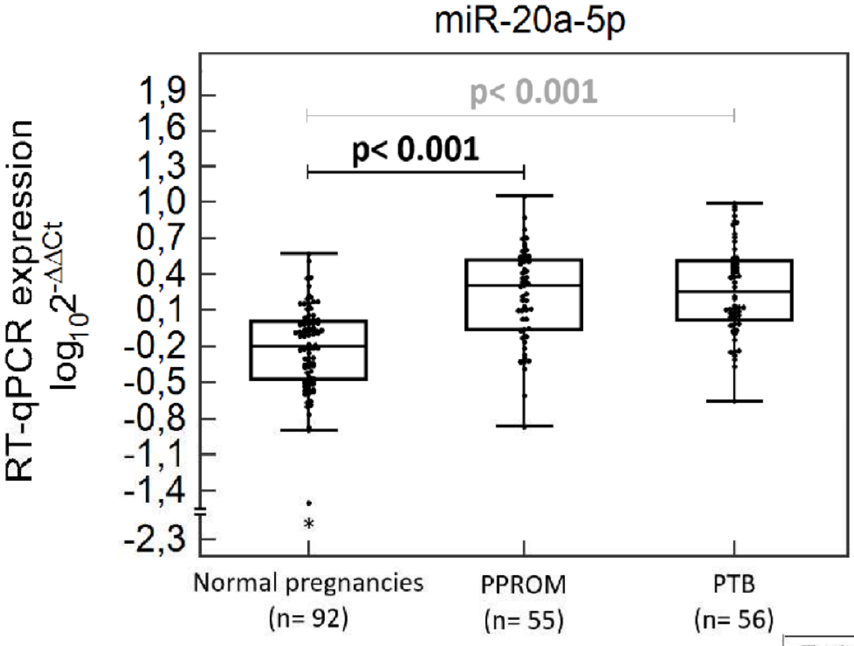

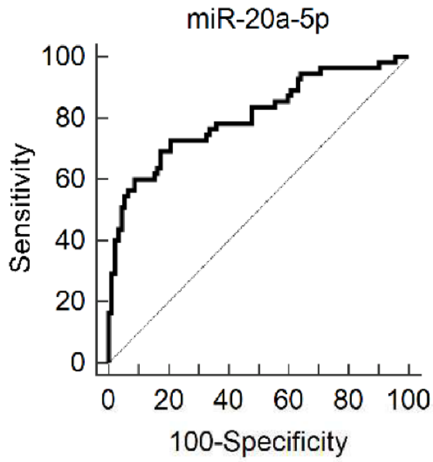

Area under the ROC curve (AUC)	0.805
Standard Error	0.0394
95% Confidence interval	0.732 to 0.866
Significance level P (Area=0.5)	<0.0001

Estimated sensitivity at fixed specificity			
Specificity	Sensitivity	95% CI	Criterion
90.00	60.00	45.45 to 74.55	>1.4726476

Criterion	Sensitivity	95% CI	Specificity	95% CI	+LR	95% CI	-LR	95% CI
>1.042907	72.73	59.0 - 83.9	79.35	69.6 - 87.1	3.52	2.3 - 5.4	0.34	0.2 - 0.5

C

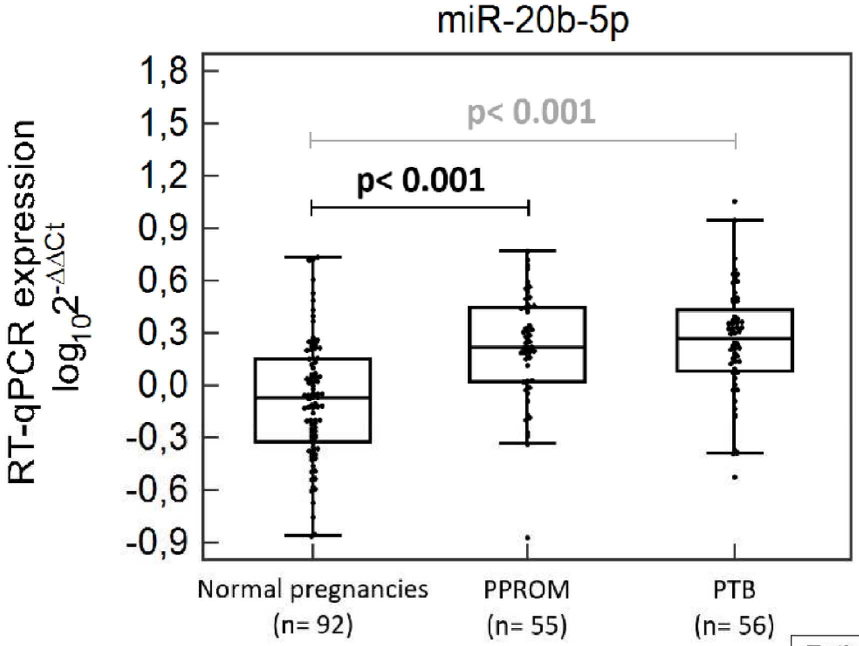

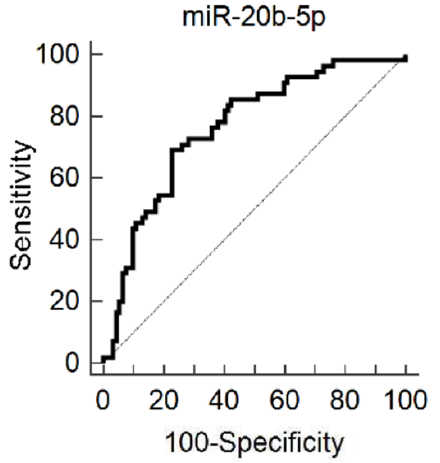

Area under the ROC curve (AUC)	0.763
Standard Error	0.0406
95% Confidence interval	0.686 to 0.829
Significance level P (Area=0.5)	<0.0001

Estimated sensitivity at fixed specificity			
Specificity	Sensitivity	95% CI	Criterion
90.00	43.64	20.00 to 64.46	>1.863984038

Criterion	Sensitivity	95% CI	Specificity	95% CI	+LR	95% CI	-LR	95% CI
>1.428041	69.09	55.2 - 80.9	77.17	67.2 - 85.3	3.03	2.0 - 4.6	0.40	0.3 - 0.6

D

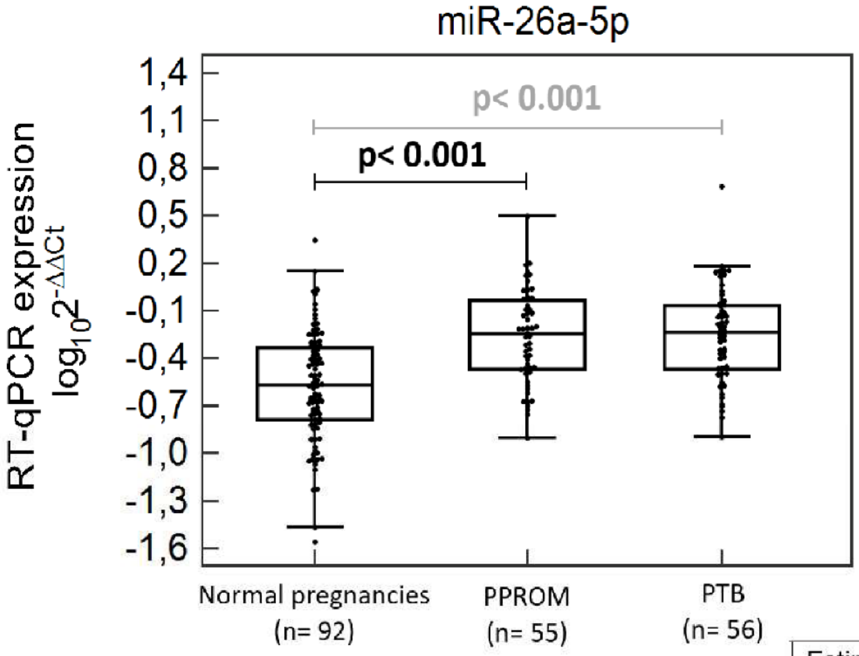

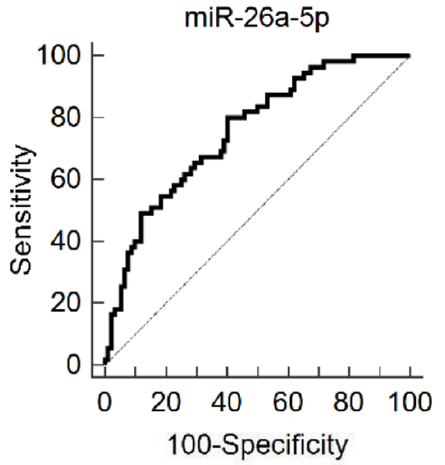

Area under the ROC curve (AUC)	0.755
Standard Error	0.0402
95% Confidence interval	0.677 to 0.822
Significance level P (Area=0.5)	<0.0001

Estimated sensitivity at fixed specificity			
Specificity	Sensitivity	95% CI	Criterion
90.00	40.00	18.18 to 60.00	>0.657045461

Criterion	Sensitivity	95% CI	Specificity	95% CI	+LR	95% CI	-LR	95% CI
>0.314884334	80.00	67.0 - 89.6	59.78	49.0 - 69.9	1.99	1.5 - 2.6	0.33	0.2 - 0.6

E

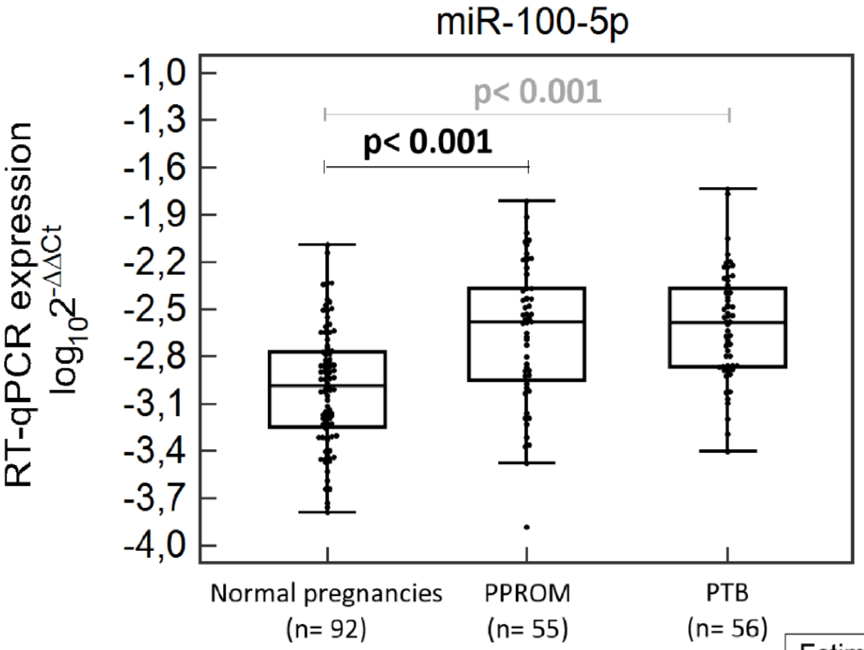

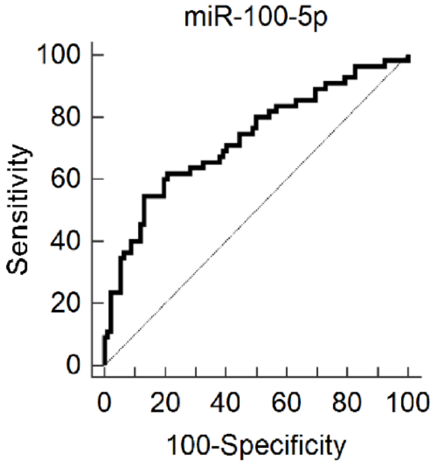

Area under the ROC curve (AUC)	0.729
Standard Error	0.0447
95% Confidence interval	0.649 to 0.799
Significance level P (Area=0.5)	<0.0001

Estimated sensitivity at fixed specificity			
Specificity	Sensitivity	95% CI	Criterion
90.00	40.00	21.82 to 63.64	>0.003099994

Criterion	Sensitivity	95% CI	Specificity	95% CI	+LR	95% CI	-LR	95% CI
>0.002524973	54.55	40.6 - 68.0	86.96	78.3 - 93.1	4.18	2.3 - 7.5	0.52	0.4 - 0.7

F

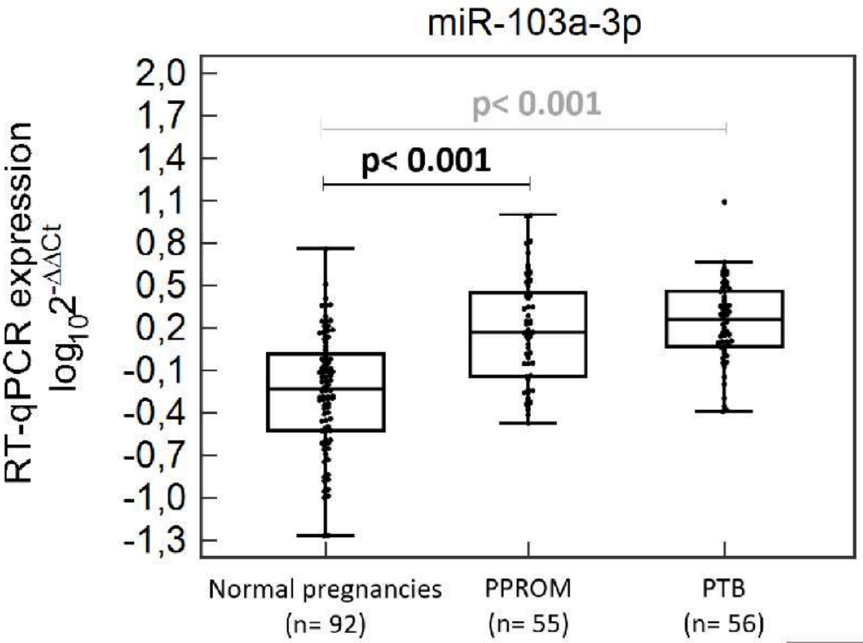

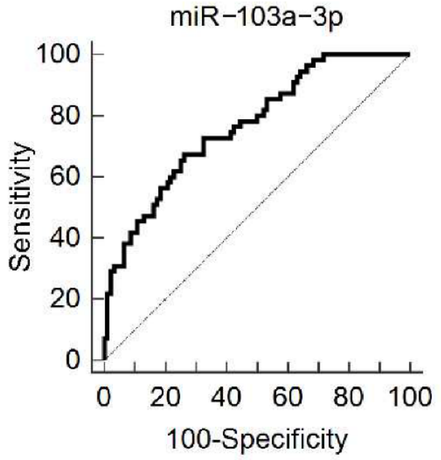

Area under the ROC curve (AUC)	0.768
Standard Error	0.0394
95% Confidence interval	0.691 to 0.833
Significance level P (Area=0.5)	<0.0001

Estimated sensitivity at fixed specificity			
Specificity	Sensitivity	95% CI	Criterion
90.00	41.82	24.23 to 56.36	>1.762985742

Criterion	Sensitivity	95% CI	Specificity	95% CI	+LR	95% CI	-LR	95% CI
>0.966205772	67.27	53.3 - 79.3	73.91	63.7 - 82.5	2.58	1.7 - 3.8	0.44	0.3 - 0.7

G

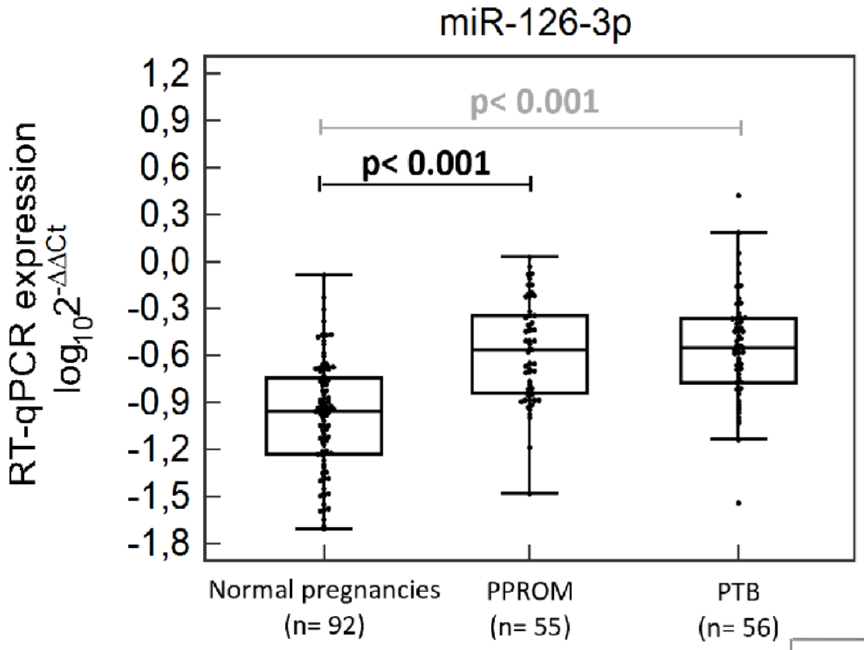

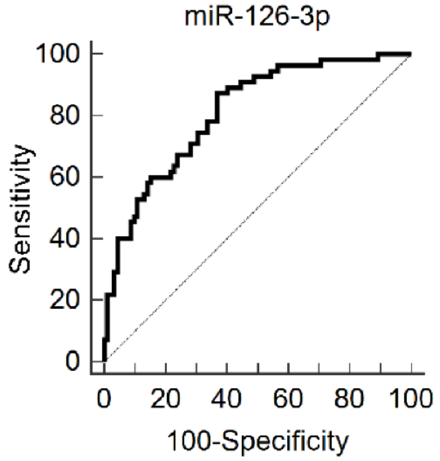

Area under the ROC curve (AUC)	0.811
Standard Error	0.0357
95% Confidence interval	0.738 to 0.871
Significance level P (Area=0.5)	<0.0001

Estimated sensitivity at fixed specificity			
Specificity	Sensitivity	95% CI	Criterion
90.00	47.27	27.27 to 63.64	>0.292364059

Criterion	Sensitivity	95% CI	Specificity	95% CI	+LR	95% CI	-LR	95% CI
>0.12652368	87.27	75.5 - 94.7	63.04	52.3 - 72.9	2.36	1.8 - 3.1	0.20	0.10 - 0.4

H

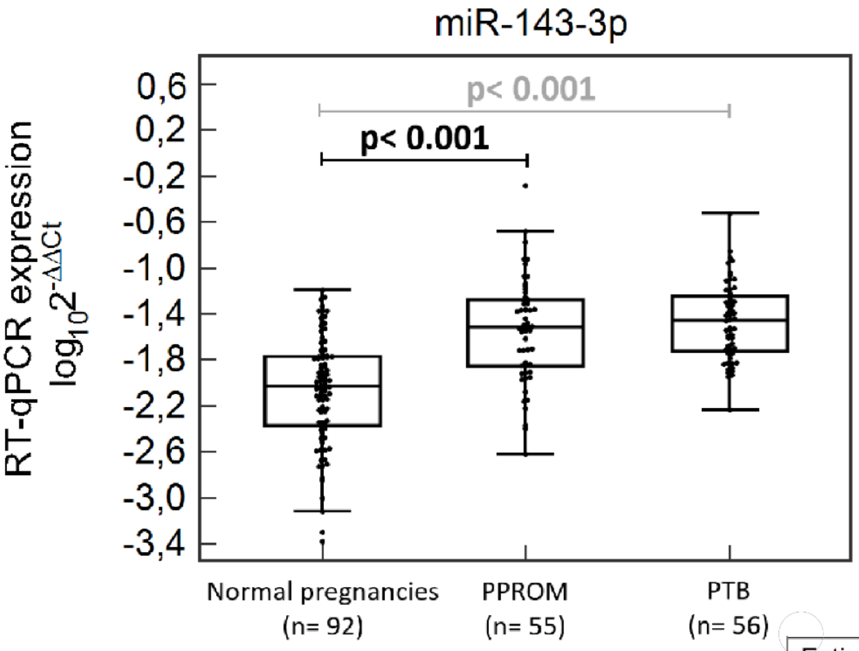

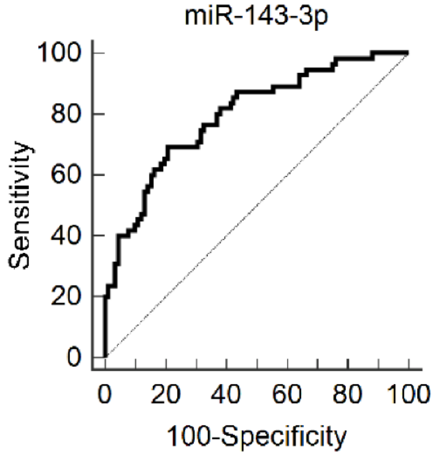

Area under the ROC curve (AUC)	0.796
Standard Error	0.0381
95% Confidence interval	0.721 to 0.858
Significance level P (Area=0.5)	<0.0001

Estimated sensitivity at fixed specificity			
Specificity	Sensitivity	95% CI	Criterion
90.00	43.64	27.27 to 65.45	>0.033743815

Criterion	Sensitivity	95% CI	Specificity	95% CI	+LR	95% CI	-LR	95% CI
>0.018386571	69.09	55.2 - 80.9	79.35	69.6 - 87.1	3.35	2.2 - 5.2	0.39	0.3 - 0.6

I

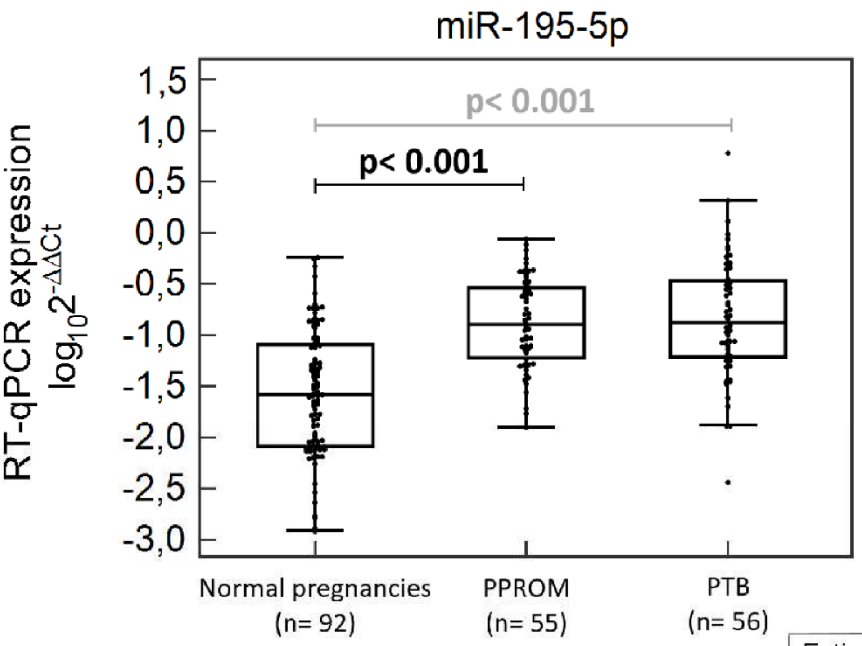

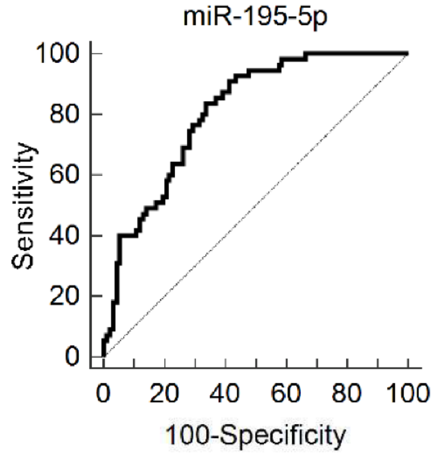

Area under the ROC curve (AUC)	0.807
Standard Error	0.0350
95% Confidence interval	0.734 to 0.867
Significance level P (Area=0.5)	<0.0001

Estimated sensitivity at fixed specificity			
Specificity	Sensitivity	95% CI	Criterion
90.00	40.00	20.00 to 54.55	>0.182605019

Criterion	Sensitivity	95% CI	Specificity	95% CI	+LR	95% CI	-LR	95% CI
>0.048301315	83.64	71.2 - 92.2	66.30	55.7 - 75.8	2.48	1.8 - 3.4	0.25	0.1 - 0.5

J

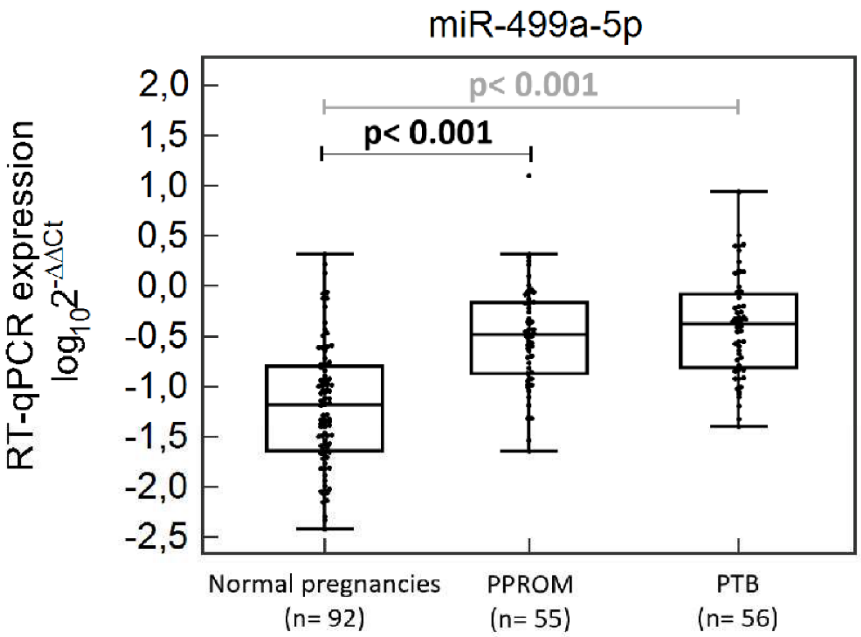

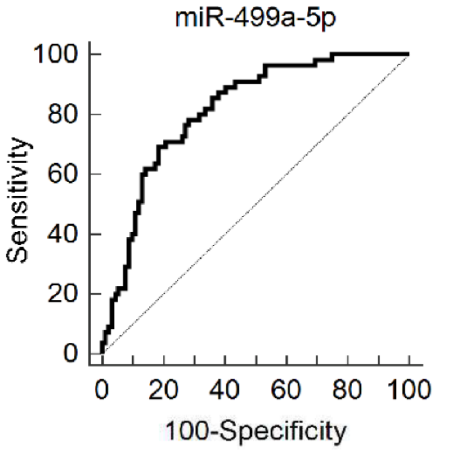

Area under the ROC curve (AUC)	0.814
Standard Error	0.0352
95% Confidence interval	0.741 to 0.873
Significance level P (Area=0.5)	<0.0001

Estimated sensitivity at fixed specificity			
Specificity	Sensitivity	95% CI	Criterion
90.00	40.00	12.31 to 67.27	>0.371976513

Criterion	Sensitivity	95% CI	Specificity	95% CI	+LR	95% CI	-LR	95% CI
>0.189959894	69.09	55.2 - 80.9	81.52	72.1 - 88.9	3.74	2.4 - 5.9	0.38	0.3 - 0.6

Supplementary Figure S1. Altered Postnatal MicroRNA Expression Profile in Children Descending from PPROM Pregnancies. Box plots and ROC curves of 10 selected individual microRNAs (miR-1-3p, miR-20a-5p, miR-20b-5p, miR-26a-5p, miR-100-5p, miR-103a-3p, miR-126-3p, miR-143-3p, miR-195-5p, and miR-499a-5p) with the best sensitivity at 10.0% FPR are presented.
