# Supplementary material for: Postnatal Expression Profile of MicroRNAs Associated with Cardiovascular Diseases in 3- to 11-Year-Old Preterm-Born Children
Source: Biomedicines. 2021 Jun 24;9(7):727. doi: 10.3390/biomedicines9070727 (PMC8301298; doi:10.3390/biomedicines9070727)
Supplement: Supplementary file 1 [file biomedicines-09-00727-s001.zip › Supplementary Figure S2.pdf]

Supplementary Figure S2.

A

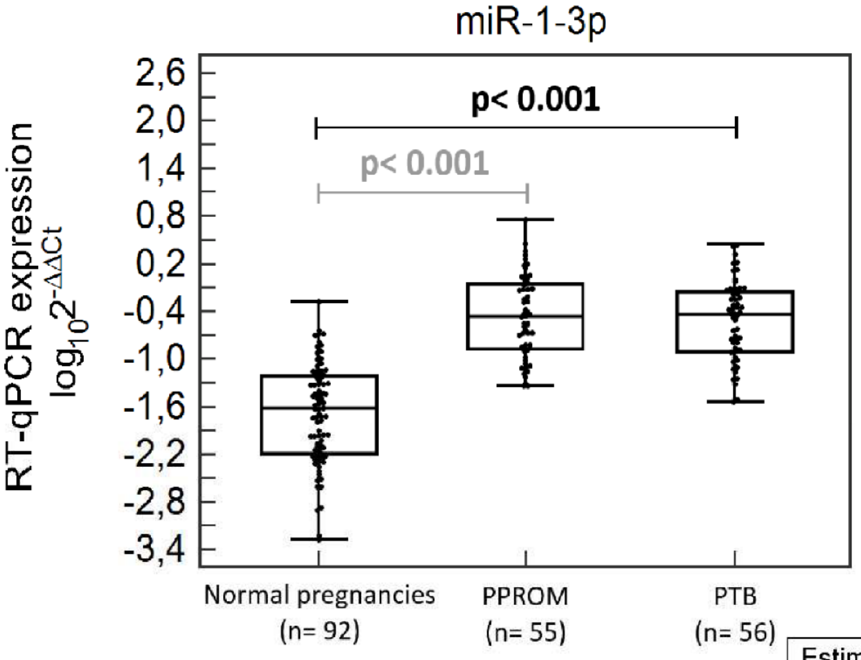

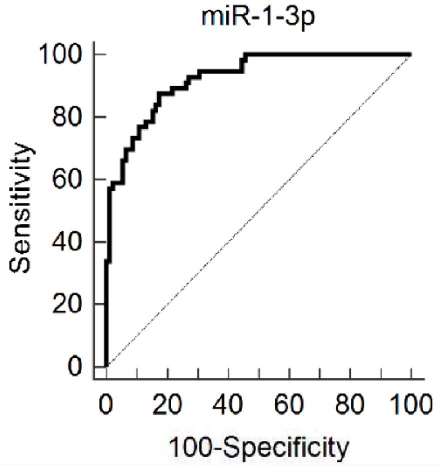

Area under the ROC curve (AUC)	0.924
Standard Error	0.0207
95% Confidence interval	0.869 to 0.961
Significance level P (Area=0.5)	<0.0001

Estimated sensitivity at fixed specificity			
Specificity	Sensitivity	95% CI	Criterion
90.00	73.21	48.21 to 83.93	>0.125703896

Criterion	Sensitivity	95% CI	Specificity	95% CI	+LR	95% CI	-LR	95% CI
>0.073354052	87.50	75.9 - 94.8	82.61	73.3 - 89.7	5.03	3.2 - 7.9	0.15	0.08 - 0.3

B

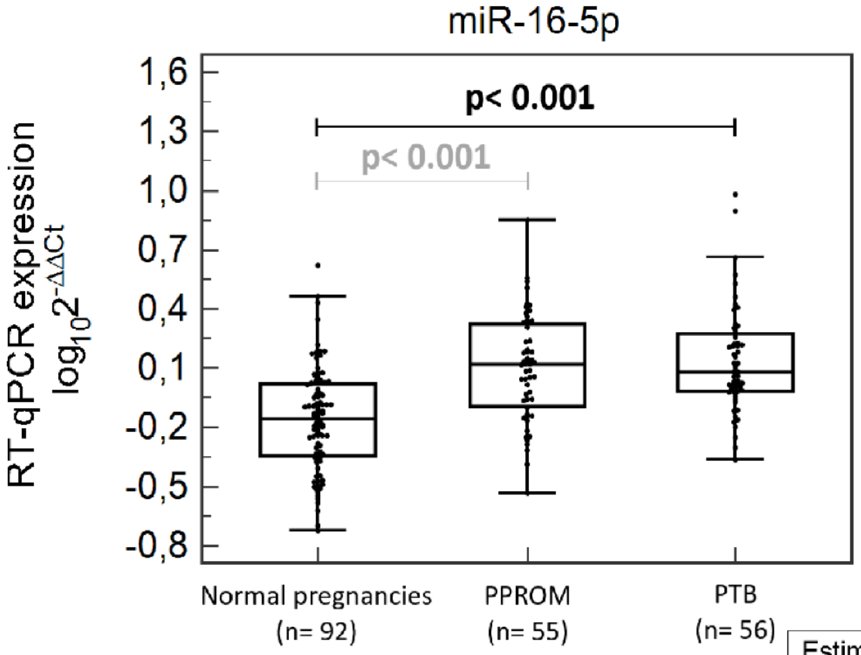

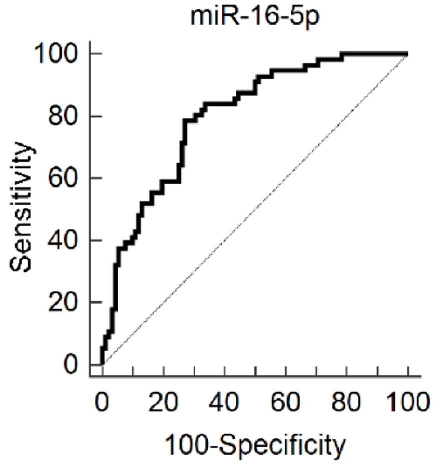

Area under the ROC curve (AUC)	0.799
Standard Error	0.0363
95% Confidence interval	0.725 to 0.860
Significance level P (Area=0.5)	<0.0001

Estimated sensitivity at fixed specificity			
Specificity	Sensitivity	95% CI	Criterion
90.00	41.07	17.86 to 57.14	>1.457966788

Criterion	Sensitivity	95% CI	Specificity	95% CI	+LR	95% CI	-LR	95% CI
>0.937039379	78.57	65.6 - 88.4	72.83	62.6 - 81.6	2.89	2.0 - 4.2	0.29	0.2 - 0.5

C

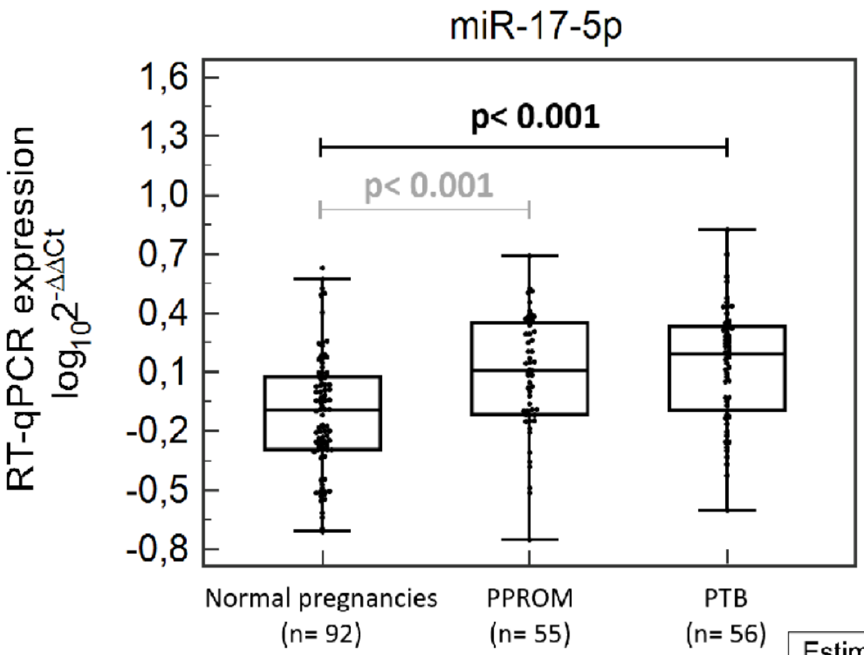

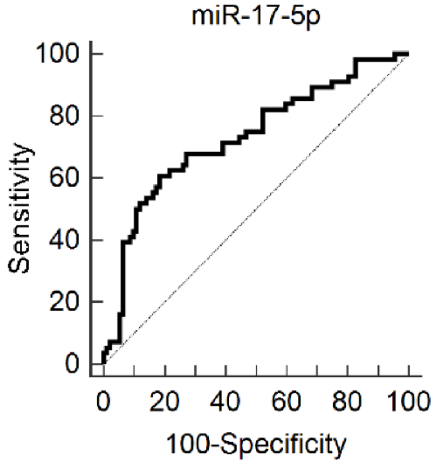

Area under the ROC curve (AUC)	0.730
Standard Error	0.0441
95% Confidence interval	0.651 to 0.800
Significance level P (Area=0.5)	<0.0001

Estimated sensitivity at fixed specificity			
Specificity	Sensitivity	95% CI	Criterion
90.00	42.86	1.79 to 57.14	>1.705247042

Criterion	Sensitivity	95% CI	Specificity	95% CI	+LR	95% CI	-LR	95% CI
>1.254276539	60.71	46.8 - 73.5	81.52	72.1 - 88.9	3.29	2.0 - 5.3	0.48	0.3 - 0.7

D

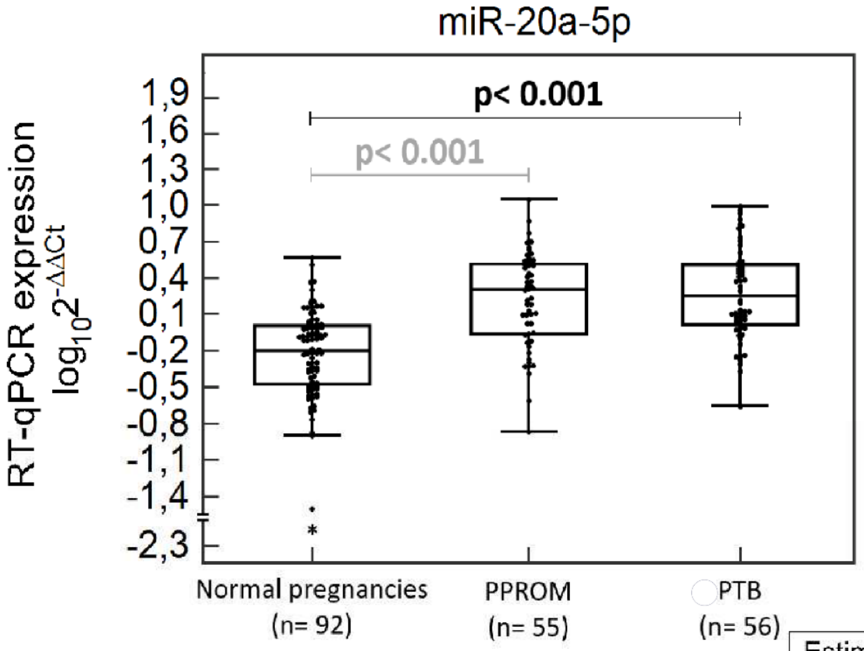

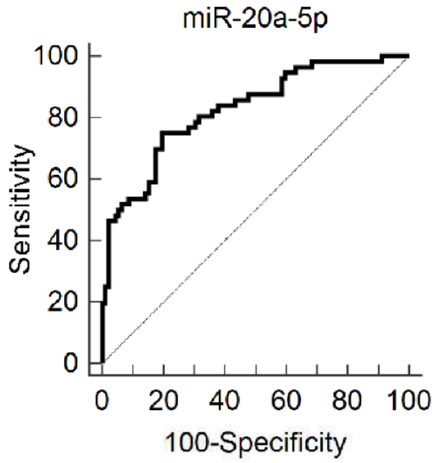

Area under the ROC curve (AUC)	0.826
Standard Error	0.0354
95% Confidence interval	0.755 to 0.883
Significance level P (Area=0.5)	<0.0001

Estimated sensitivity at fixed specificity			
Specificity	Sensitivity	95% CI	Criterion
90.00	53.57	39.29 to 67.86	>1.4726476

Criterion	Sensitivity	95% CI	Specificity	95% CI	+LR	95% CI	-LR	95% CI
>1.096585	75.00	61.6 - 85.6	80.43	70.9 - 88.0	3.83	2.5 - 6.0	0.31	0.2 - 0.5

E

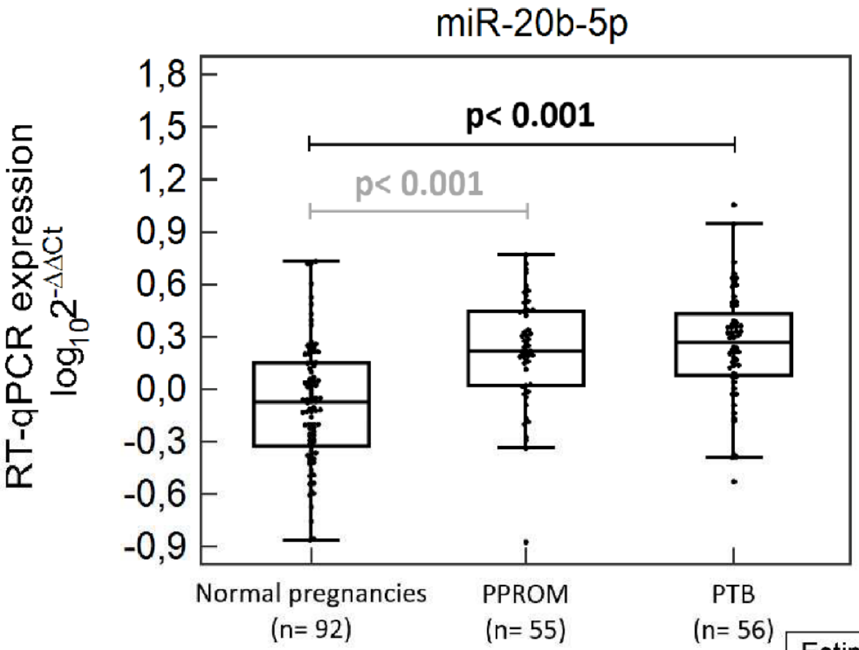

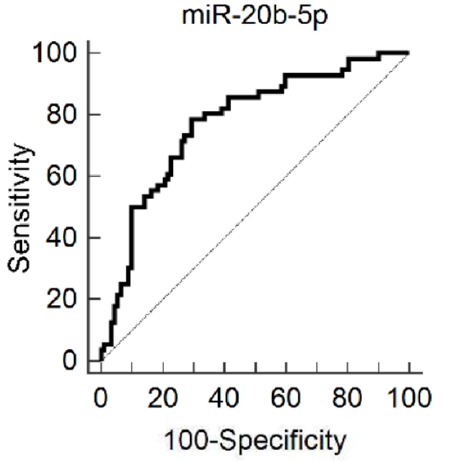

Area under the ROC curve (AUC)	0.774
Standard Error	0.0398
95% Confidence interval	0.698 to 0.839
Significance level P (Area=0.5)	<0.0001

Estimated sensitivity at fixed specificity			
Specificity	Sensitivity	95% CI	Criterion
90.00	50.00	21.43 to 67.86	>1.8604736

Criterion	Sensitivity	95% CI	Specificity	95% CI	+LR	95% CI	-LR	95% CI
>1.16812	78.57	65.6 - 88.4	70.65	60.2 - 79.7	2.68	1.9 - 3.8	0.30	0.2 - 0.5

F

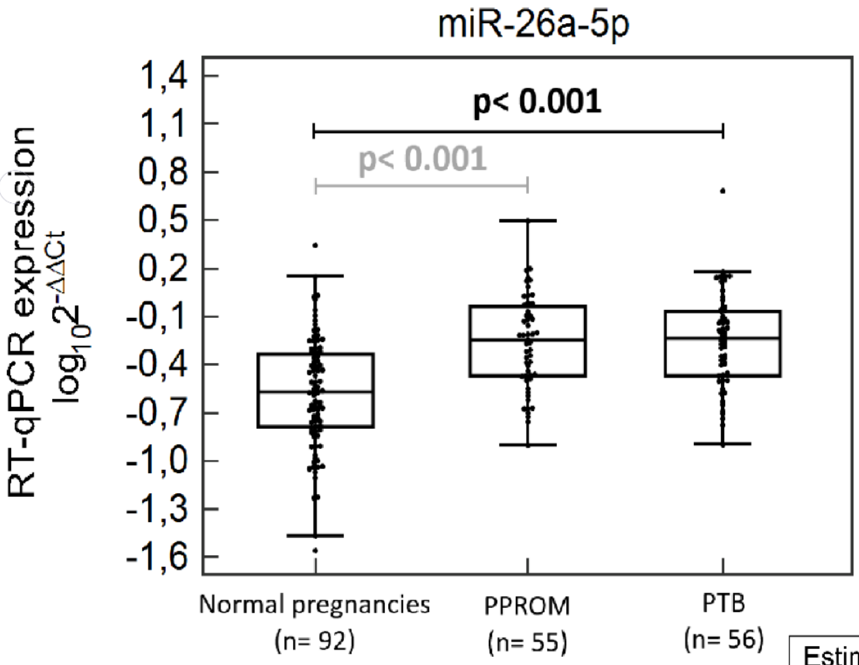

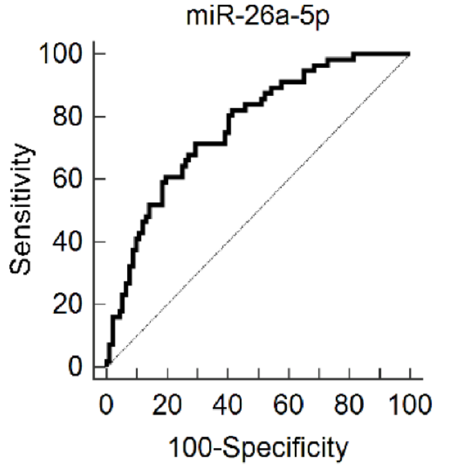

Area under the ROC curve (AUC)	0.767
Standard Error	0.0391
95% Confidence interval	0.691 to 0.833
Significance level P (Area=0.5)	<0.0001

Estimated sensitivity at fixed specificity			
Specificity	Sensitivity	95% CI	Criterion
90.00	41.07	16.07 to 59.56	>0.65754368

Criterion	Sensitivity	95% CI	Specificity	95% CI	+LR	95% CI	-LR	95% CI
>0.394435839	71.43	57.8 - 82.7	70.65	60.2 - 79.7	2.43	1.7 - 3.5	0.40	0.3 - 0.6

G

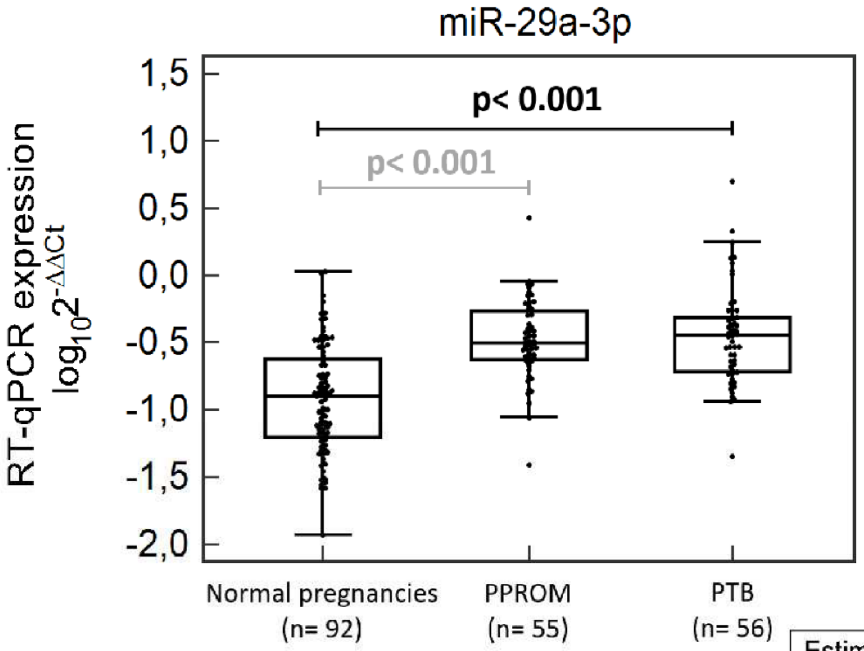

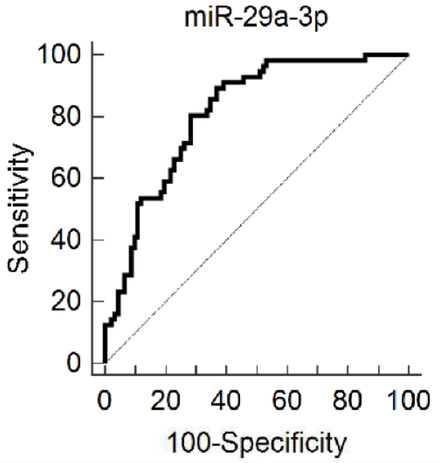

Area under the ROC curve (AUC)	0.812
Standard Error	0.0349
95% Confidence interval	0.740 to 0.872
Significance level P (Area=0.5)	<0.0001

Estimated sensitivity at fixed specificity			
Specificity	Sensitivity	95% CI	Criterion
90.00	41.07	12.50 to 60.71	>0.385266192

Criterion	Sensitivity	95% CI	Specificity	95% CI	+LR	95% CI	-LR	95% CI
>0.147129196	89.29	78.1 - 96.0	63.04	52.3 - 72.9	2.42	1.8 - 3.2	0.17	0.08 - 0.4

H

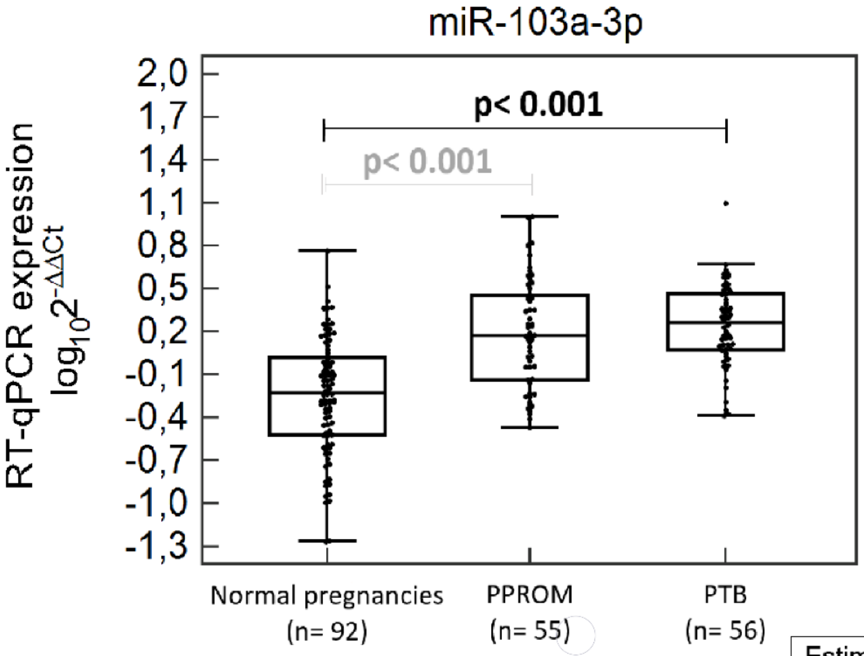

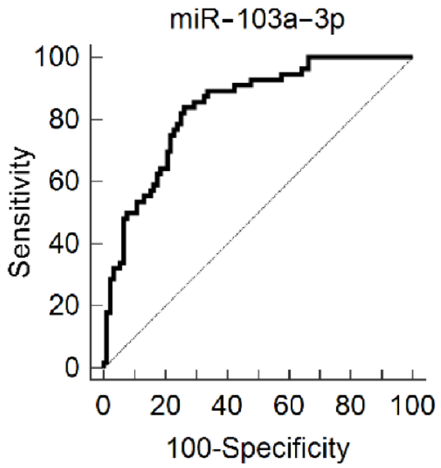

Area under the ROC curve (AUC)	0.839
Standard Error	0.0326
95% Confidence interval	0.769 to 0.894
Significance level P (Area=0.5)	<0.0001

Estimated sensitivity at fixed specificity			
Specificity	Sensitivity	95% CI	Criterion
90.00	50.00	22.06 to 62.50	>1.765207853

Criterion	Sensitivity	95% CI	Specificity	95% CI	+LR	95% CI	-LR	95% CI
>0.966205772	83.93	71.7 - 92.4	73.91	63.7 - 82.5	3.22	2.2 - 4.6	0.22	0.1 - 0.4

I

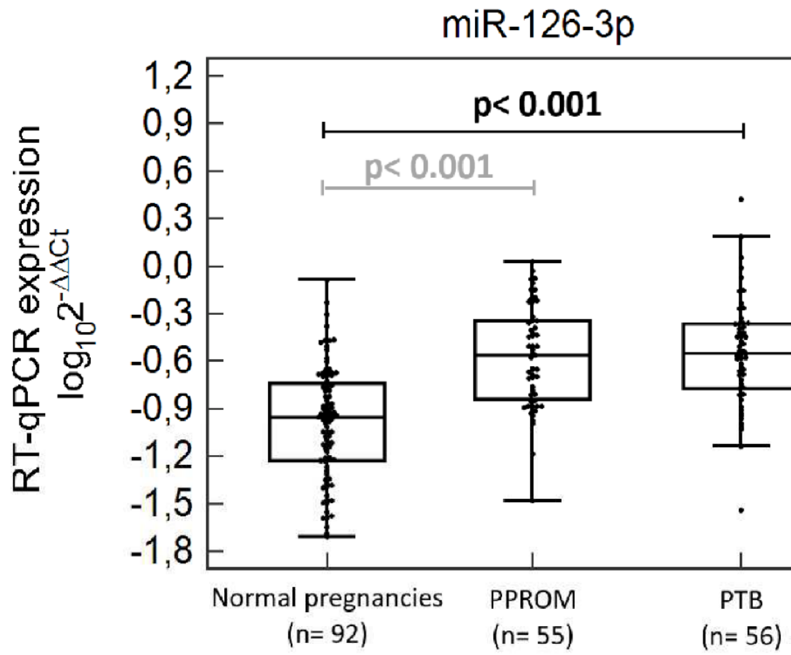

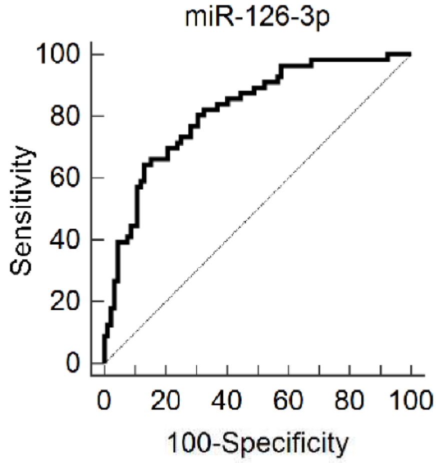

Area under the ROC curve (AUC)	0.818
Standard Error	0.0355
95% Confidence interval	0.747 to 0.877
Significance level P (Area=0.5)	<0.0001

Estimated sensitivity at fixed specificity

Specificity	Sensitivity	95% CI	Criterion
90.00	44.64	17.91 to 64.29	>0.295345179

Criterion	Sensitivity	95% CI	Specificity	95% CI	+LR	95% CI	-LR	95% CI
>0.221806381	64.29	50.4 - 76.6	86.96	78.3 - 93.1	4.93	2.8 - 8.7	0.41	0.3 - 0.6

J

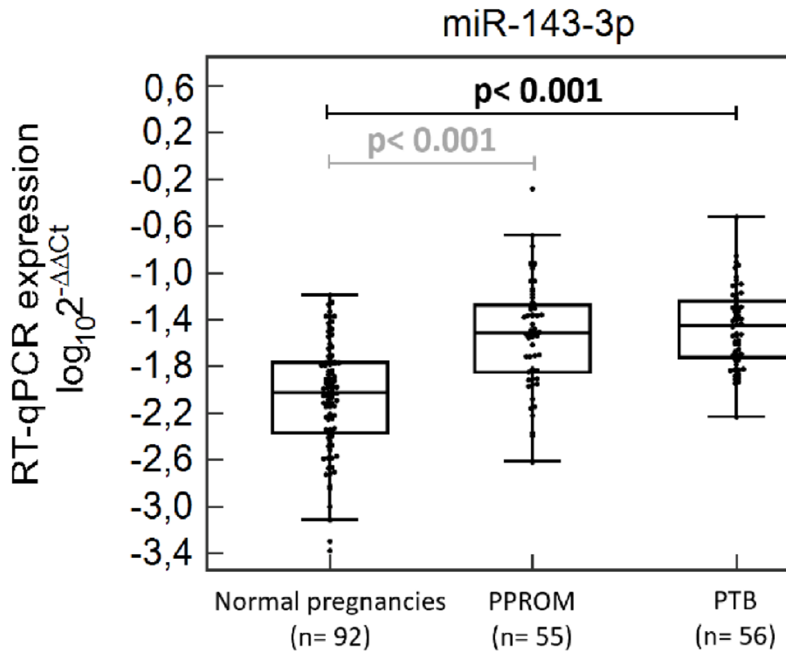

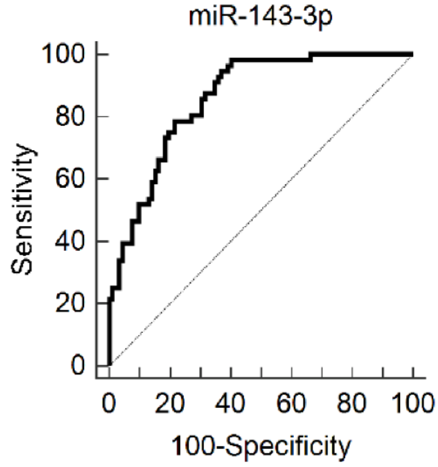

Area under the ROC curve (AUC)	0.860
Standard Error	0.0293
95% Confidence interval	0.793 to 0.911
Significance level P (Area=0.5)	<0.0001

Estimated sensitivity at fixed specificity

Specificity	Sensitivity	95% CI	Criterion
90.00	51.79	33.93 to 73.21	>0.033723214

Criterion	Sensitivity	95% CI	Specificity	95% CI	+LR	95% CI	-LR	95% CI
>0.011410549	98.21	90.4 - 100.0	59.78	49.0 - 69.9	2.44	1.9 - 3.1	0.030	0.004 - 0.2

K

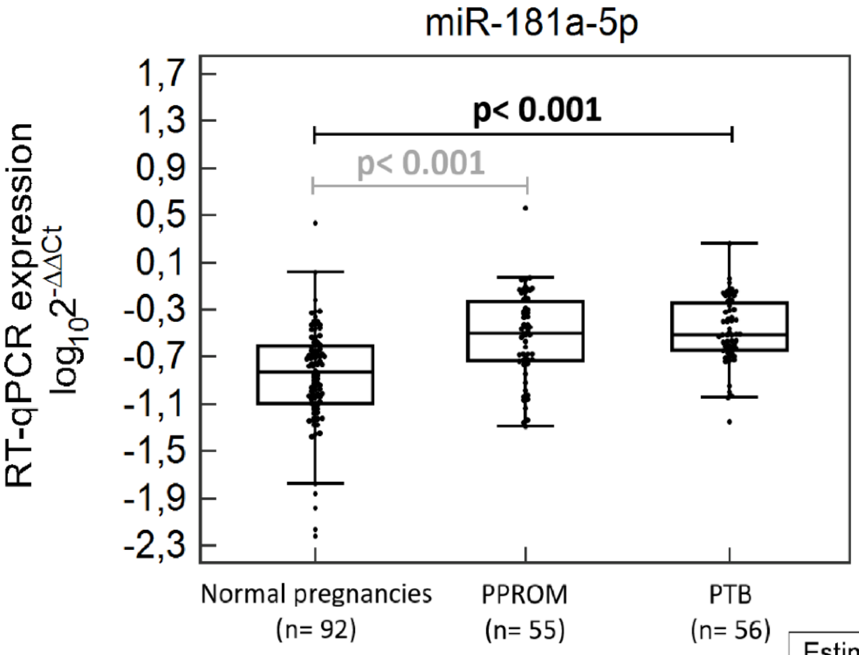

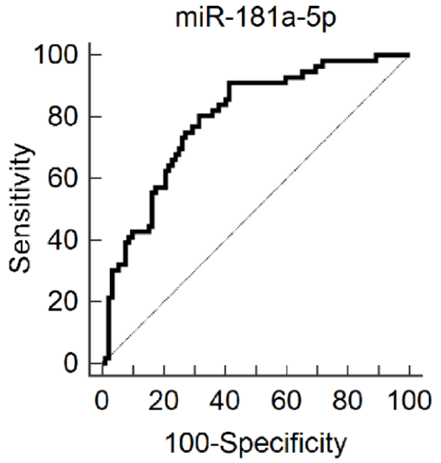

Area under the ROC curve (AUC)	0.795
Standard Error	0.0372
95% Confidence interval	0.721 to 0.857
Significance level P (Area=0.5)	<0.0001

Estimated sensitivity at fixed specificity

Specificity	Sensitivity	95% CI	Criterion
90.00	42.86	28.57 to 60.71	>0.380849491

Criterion	Sensitivity	95% CI	Specificity	95% CI	+LR	95% CI	-LR	95% CI
>0.18456279	91.07	80.4 - 97.0	58.70	47.9 - 68.9	2.20	1.7 - 2.9	0.15	0.06 - 0.4

L

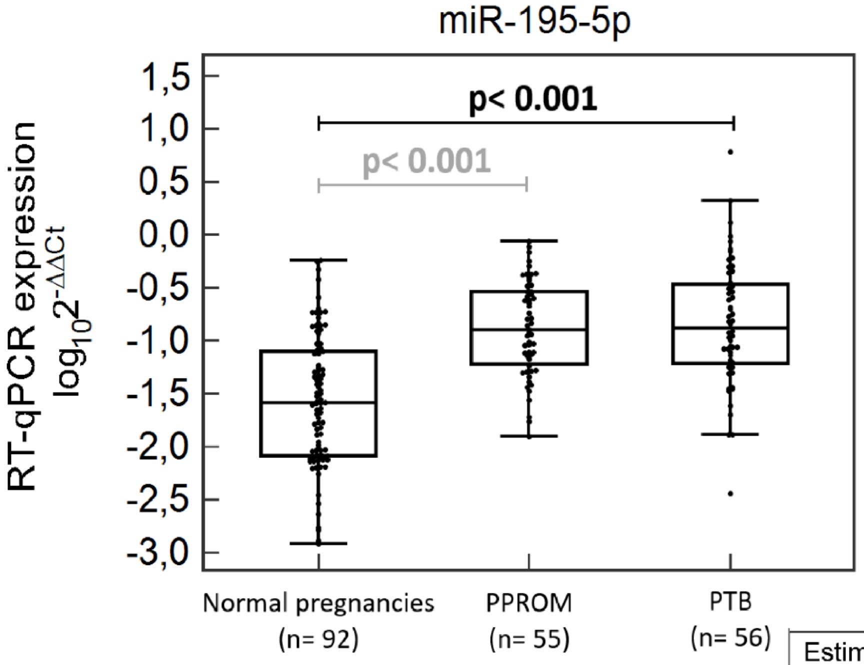

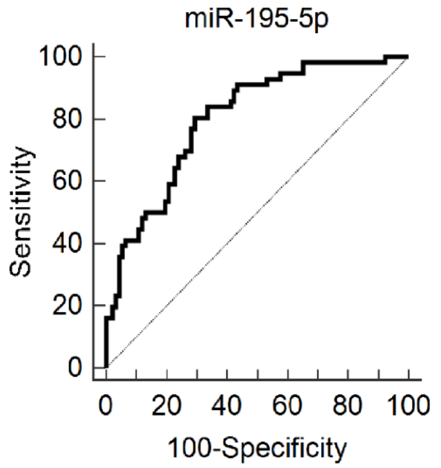

Area under the ROC curve (AUC)	0.803
Standard Error	0.0362
95% Confidence interval	0.730 to 0.864
Significance level P (Area=0.5)	<0.0001

Estimated sensitivity at fixed specificity

Specificity	Sensitivity	95% CI	Criterion
90.00	41.07	19.43 to 53.57	>0.181981892

Criterion	Sensitivity	95% CI	Specificity	95% CI	+LR	95% CI	-LR	95% CI
>0.055341746	80.36	67.6 - 89.8	70.65	60.2 - 79.7	2.74	1.9 - 3.9	0.28	0.2 - 0.5

M

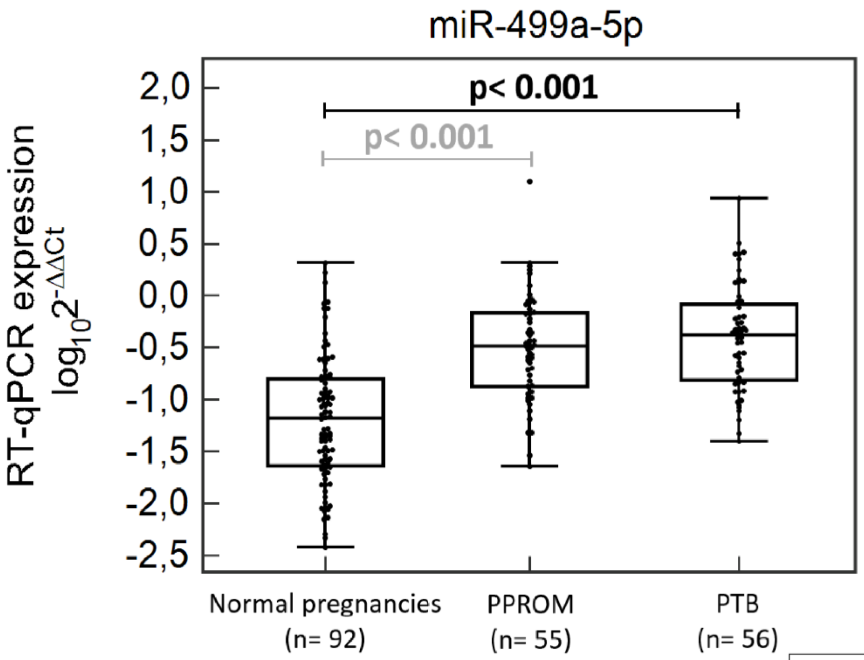

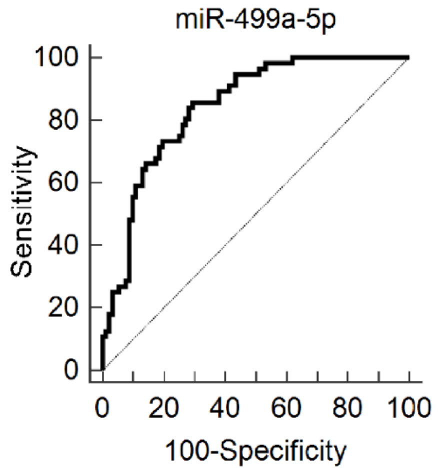

Area under the ROC curve (AUC)	0.843
Standard Error	0.0316
95% Confidence interval	0.774 to 0.898
Significance level P (Area=0.5)	<0.0001

Estimated sensitivity at fixed specificity			
Specificity	Sensitivity	95% CI	Criterion
90.00	55.36	19.64 to 73.21	>0.36883257

Criterion	Sensitivity	95% CI	Specificity	95% CI	+LR	95% CI	-LR	95% CI
>0.11803281	85.71	73.8 - 93.6	70.65	60.2 - 79.7	2.92	2.1 - 4.1	0.20	0.1 - 0.4

Supplementary Figure S2. Altered Postnatal MicroRNA Expression Profile in Children Descending from PTB Pregnancies. Box plots and ROC curves of 13 selected individual microRNAs (miR-1-3p, miR-16-5p, miR-17-5p, miR-20a-5p, miR-20b-5p, miR-26a-5p, miR-29a-3p, miR-103a-3p, miR-126-3p, miR-143-3p, miR-181a-5p, miR-195-5p, and miR-499a-5p) with the best sensitivity at 10.0% FPR are presented.
