# Supplementary material for: Postnatal Expression Profile of MicroRNAs Associated with Cardiovascular Diseases in 3- to 11-Year-Old Preterm-Born Children
Source: Biomedicines. 2021 Jun 24;9(7):727. doi: 10.3390/biomedicines9070727 (PMC8301298; doi:10.3390/biomedicines9070727)
Supplement: Supplementary file 1 [file biomedicines-09-00727-s001.zip › Supplementary Figure S3.pdf]

Supplementary Figure S3.

A

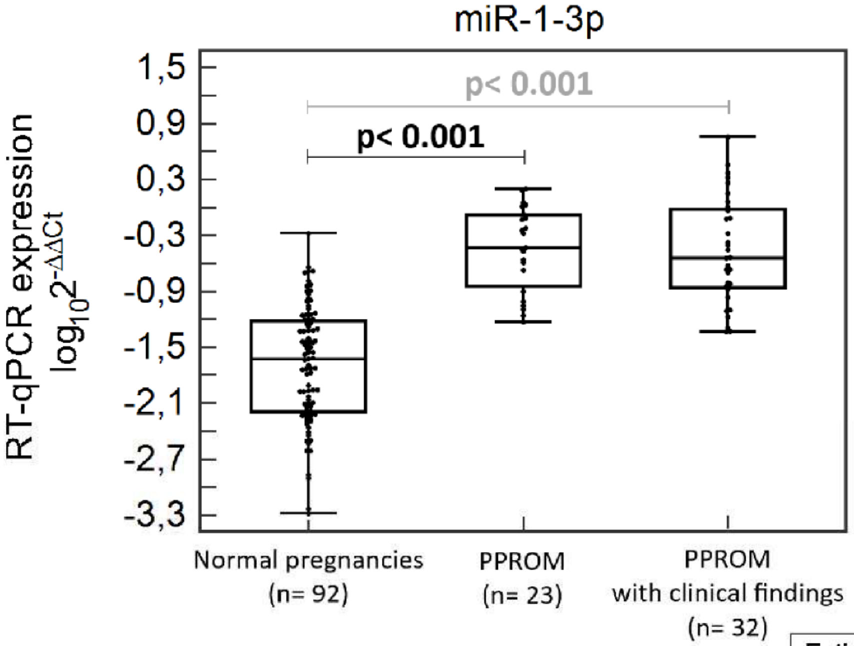

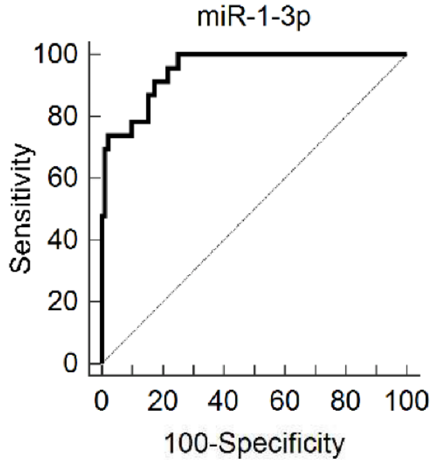

Area under the ROC curve (AUC)	0.951
Standard Error	0.0198
95% Confidence interval	0.895 to 0.983
Significance level P (Area=0.5)	<0.0001

Estimated sensitivity at fixed specificity			
Specificity	Sensitivity	95% CI	Criterion
90.00	78.26	56.52 to 95.65	>0.124151746

Criterion	Sensitivity	95% CI	Specificity	95% CI	+LR	95% CI	-LR	95% CI
>0.057714675	100.00	85.2 - 100.0	75.00	64.9 - 83.4	4.00	2.8 - 5.7	0.00	

B

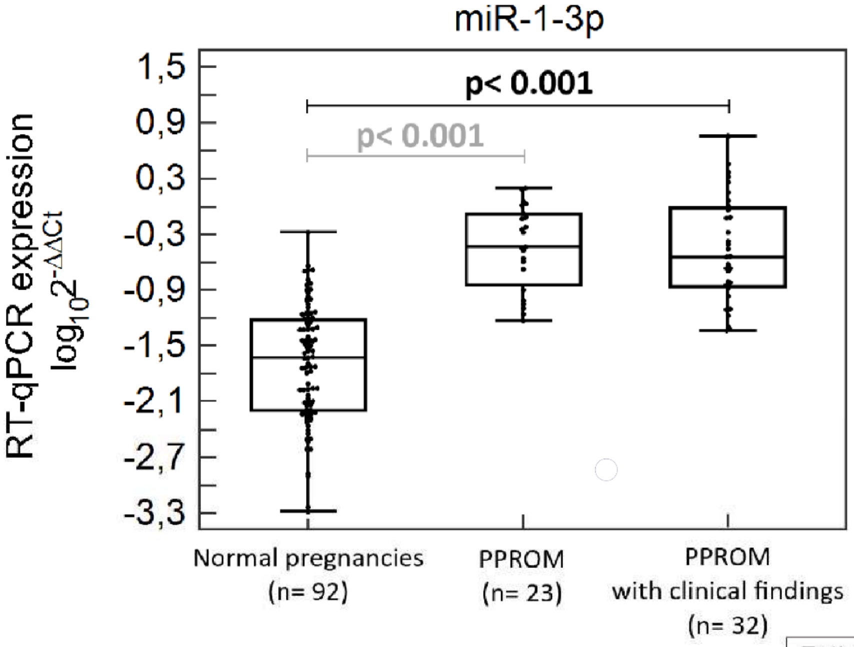

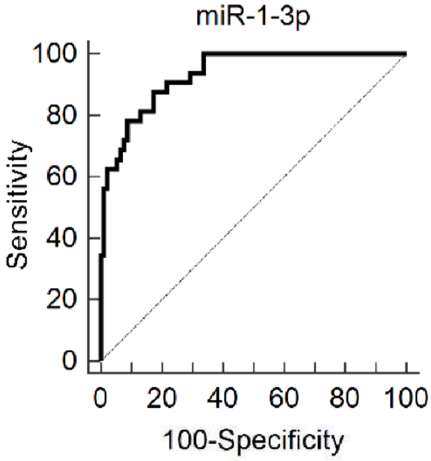

Area under the ROC curve (AUC)	0.933
Standard Error	0.0222
95% Confidence interval	0.873 to 0.970
Significance level P (Area=0.5)	<0.0001

Estimated sensitivity at fixed specificity			
Specificity	Sensitivity	95% CI	Criterion
90.00	78.12	56.25 to 90.62	>0.124151746

Criterion	Sensitivity	95% CI	Specificity	95% CI	+LR	95% CI	-LR	95% CI
>0.073354052	87.50	71.0 - 96.5	82.61	73.3 - 89.7	5.03	3.2 - 8.0	0.15	0.06 - 0.4

C

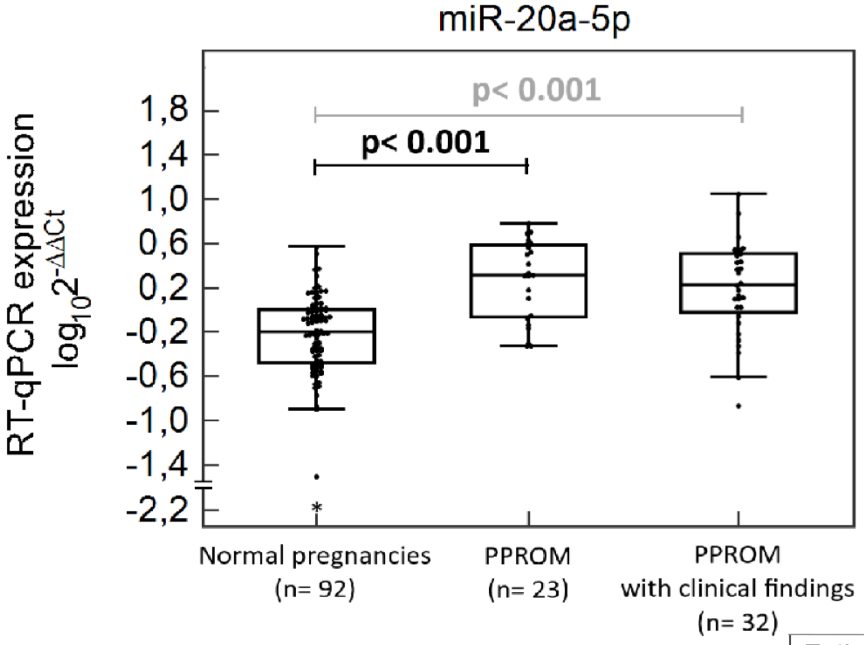

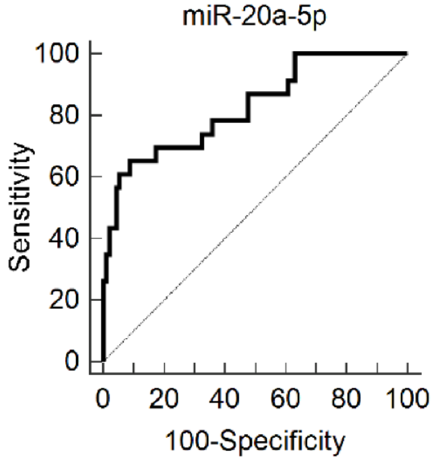

Area under the ROC curve (AUC)	0.825
Standard Error	0.0519
95% Confidence interval	0.743 to 0.890
Significance level P (Area=0.5)	<0.0001

Estimated sensitivity at fixed specificity			
Specificity	Sensitivity	95% CI	Criterion
90.00	65.22	43.48 to 82.61	>1.4726476

Criterion	Sensitivity	95% CI	Specificity	95% CI	+LR	95% CI	-LR	95% CI
>1.482506	65.22	42.7 - 83.6	91.30	83.6 - 96.2	7.50	3.6 - 15.5	0.38	0.2 - 0.7

D

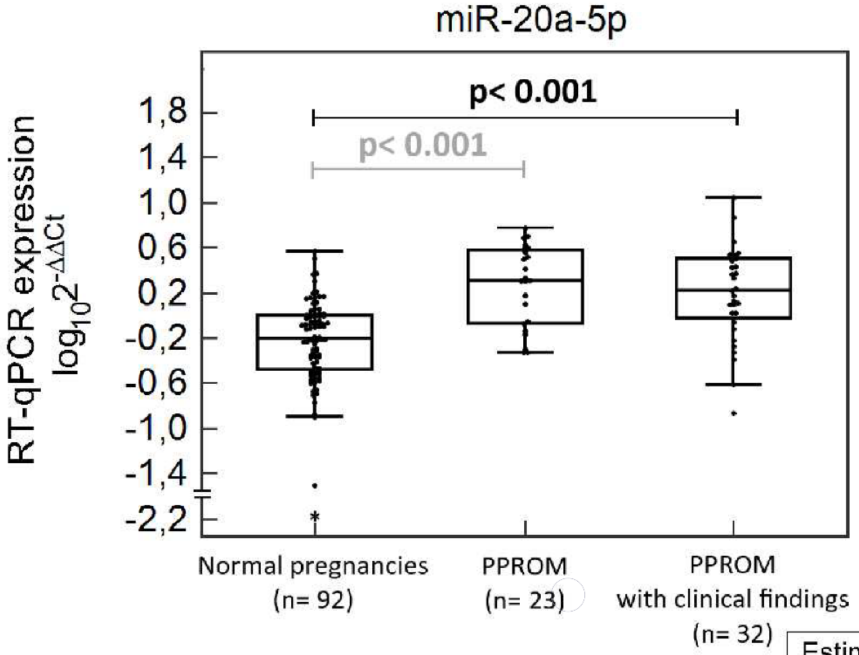

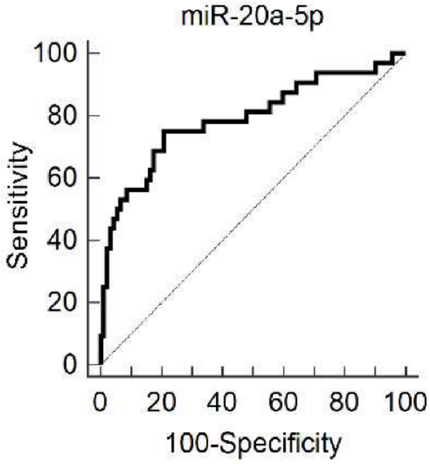

Area under the ROC curve (AUC)	0.790
Standard Error	0.0529
95% Confidence interval	0.708 to 0.858
Significance level P (Area=0.5)	<0.0001

Estimated sensitivity at fixed specificity			
Specificity	Sensitivity	95% CI	Criterion
90.00	56.25	40.63 to 75.00	>1.4726476

Criterion	Sensitivity	95% CI	Specificity	95% CI	+LR	95% CI	-LR	95% CI
>1.042907	75.00	56.6 - 88.5	79.35	69.6 - 87.1	3.63	2.3 - 5.7	0.32	0.2 - 0.6

Supplementary Figure S3. Altered Postnatal MicroRNA Expression Profile in Preterm Born Children (PPROM) with regard to the Absence or Presence of Abnormal Clinical Findings (Overweight/Obesity, Prehypertension/Hypertension, Valve Problems and Heart Defects). Box plots and ROC curves of 2 selected individual microRNAs (miR-1-3p and miR-20a-5p) with the best sensitivity at 10.0% FPR are presented.
