# Supplementary material for: Postnatal Expression Profile of MicroRNAs Associated with Cardiovascular Diseases in 3- to 11-Year-Old Preterm-Born Children
Source: Biomedicines. 2021 Jun 24;9(7):727. doi: 10.3390/biomedicines9070727 (PMC8301298; doi:10.3390/biomedicines9070727)
Supplement: Supplementary file 1 [file biomedicines-09-00727-s001.zip › Supplementary Figure S4.pdf]

Supplementary Figure S4.

A

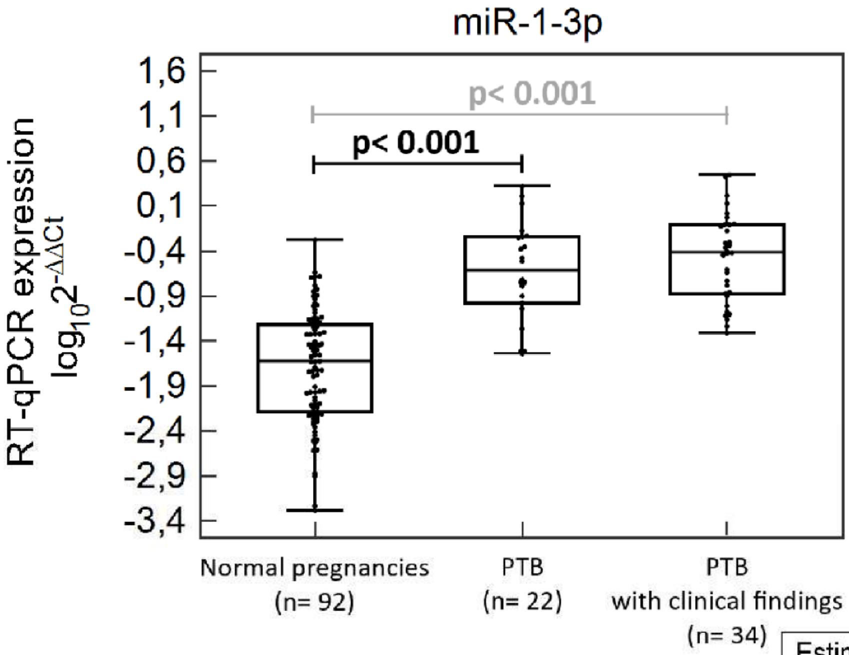

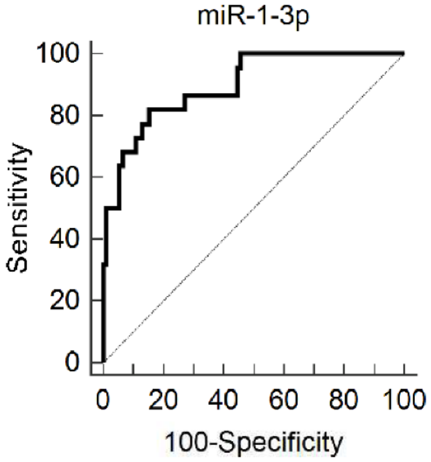

Area under the ROC curve (AUC)	0.896
Standard Error	0.0365
95% Confidence interval	0.825 to 0.946
Significance level P (Area=0.5)	<0.0001

Estimated sensitivity at fixed specificity			
Specificity	Sensitivity	95% CI	Criterion
90.00	68.18	39.55 to 86.36	>0.125703896

Criterion	Sensitivity	95% CI	Specificity	95% CI	+LR	95% CI	-LR	95% CI
>0.088522793	81.82	59.7 - 94.8	84.78	75.8 - 91.4	5.38	3.2 - 9.1	0.21	0.09 - 0.5

B

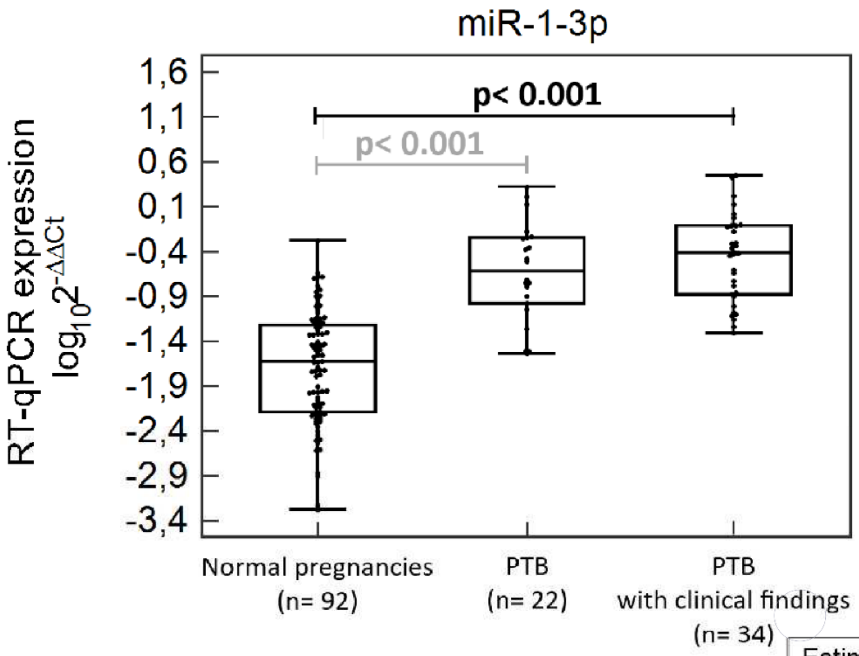

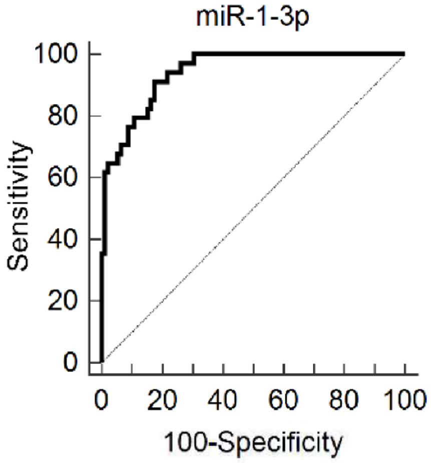

Area under the ROC curve (AUC)	0.942
Standard Error	0.0192
95% Confidence interval	0.886 to 0.976
Significance level P (Area=0.5)	<0.0001

Estimated sensitivity at fixed specificity			
Specificity	Sensitivity	95% CI	Criterion
90.00	76.47	52.94 to 91.18	>0.125174926

Criterion	Sensitivity	95% CI	Specificity	95% CI	+LR	95% CI	-LR	95% CI
>0.073354052	91.18	76.3 - 98.1	82.61	73.3 - 89.7	5.24	3.3 - 8.3	0.11	0.04 - 0.3

C

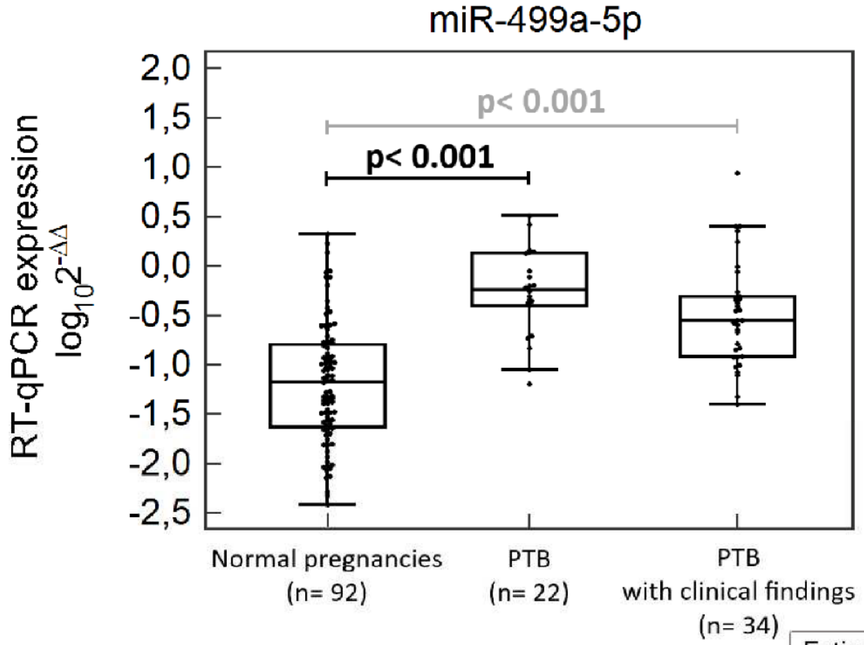

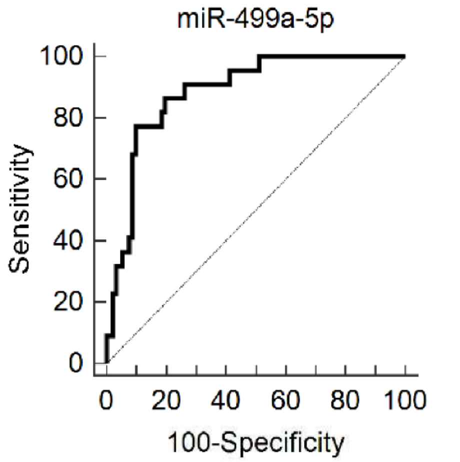

Area under the ROC curve (AUC)	0.884
Standard Error	0.0351
95% Confidence interval	0.811 to 0.937
Significance level P (Area=0.5)	<0.0001

Estimated sensitivity at fixed specificity			
Specificity	Sensitivity	95% CI	Criterion
90.00	77.27	36.36 to 100.00	>0.365652056

Criterion	Sensitivity	95% CI	Specificity	95% CI	+LR	95% CI	-LR	95% CI
>0.372468181	77.27	54.6 - 92.2	90.22	82.2 - 95.4	7.90	4.1 - 15.3	0.25	0.1 - 0.5

D

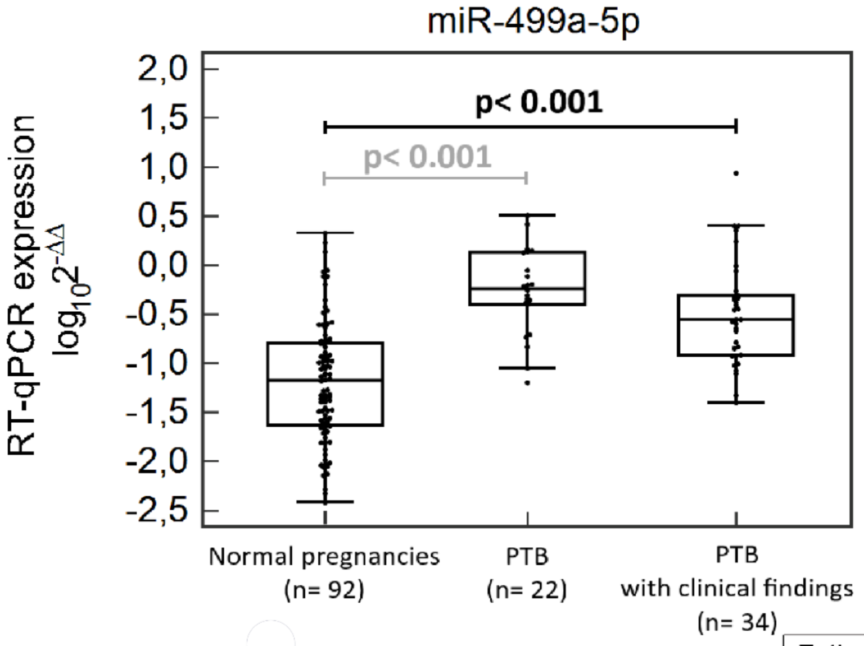

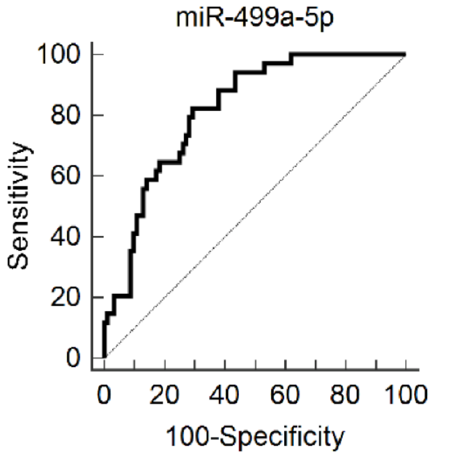

Area under the ROC curve (AUC)	0.816
Standard Error	0.0383
95% Confidence interval	0.738 to 0.880
Significance level P (Area=0.5)	<0.0001

Estimated sensitivity at fixed specificity			
Specificity	Sensitivity	95% CI	Criterion
90.00	41.18	11.76 to 67.65	>0.36883257

Criterion	Sensitivity	95% CI	Specificity	95% CI	+LR	95% CI	-LR	95% CI
>0.11803281	82.35	65.5 - 93.2	70.65	60.2 - 79.7	2.81	2.0 - 4.0	0.25	0.1 - 0.5

Supplementary Figure S4. Altered Postnatal MicroRNA Expression Profile in Preterm Born Children (PTB) with regard to the Absence or Presence of Abnormal Clinical Findings (Overweight/Obesity, Prehypertension/Hypertension, Valve Problems and Heart Defects). Box plots and ROC curves of 2 selected individual microRNAs (miR-1-3p and miR-499a-5p) with the best sensitivity at 10.0% FPR are presented.
