# Supplementary material for: Postnatal Expression Profile of MicroRNAs Associated with Cardiovascular Diseases in 3- to 11-Year-Old Preterm-Born Children
Source: Biomedicines. 2021 Jun 24;9(7):727. doi: 10.3390/biomedicines9070727 (PMC8301298; doi:10.3390/biomedicines9070727)
Supplement: Supplementary file 1 [file biomedicines-09-00727-s001.zip › Supplementary Figure S5.pdf]

Supplementary Figure S5.

A

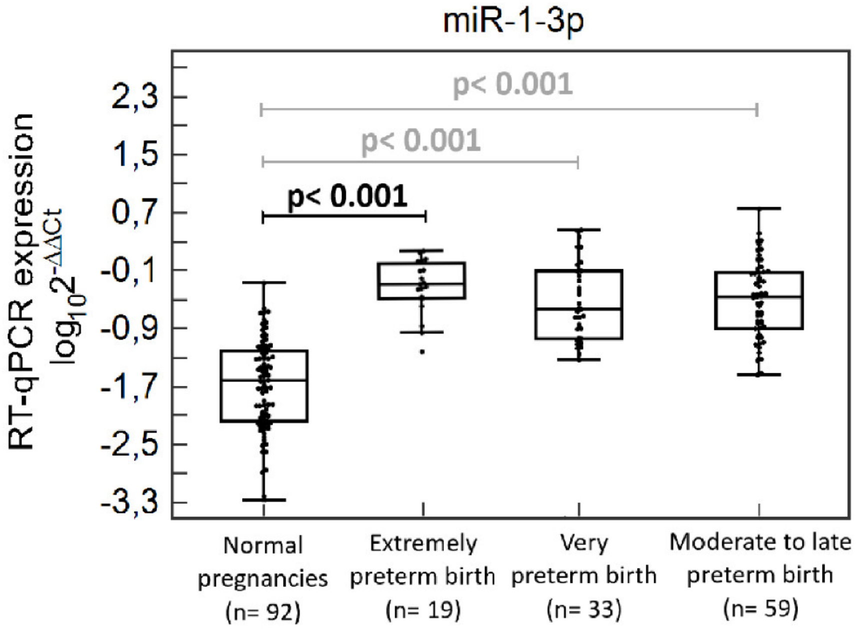

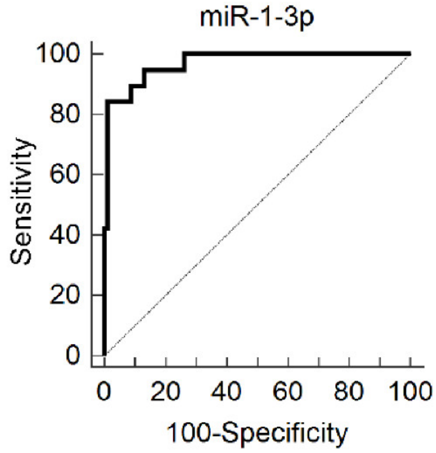

Area under the ROC curve (AUC)	0.970
Standard Error	0.0169
95% Confidence interval	0.919 to 0.993
Significance level P (Area=0.5)	<0.0001

Estimated sensitivity at fixed specificity			
Specificity	Sensitivity	95% CI	Criterion
90.00	89.47	63.16 to 100.00	>0.124151746

Criterion	Sensitivity	95% CI	Specificity	95% CI	+LR	95% CI	-LR	95% CI
>0.230010683	84.21	60.4 - 96.6	98.91	94.1 - 100.0	77.47	10.9 - 549.5	0.16	0.06 - 0.5

B

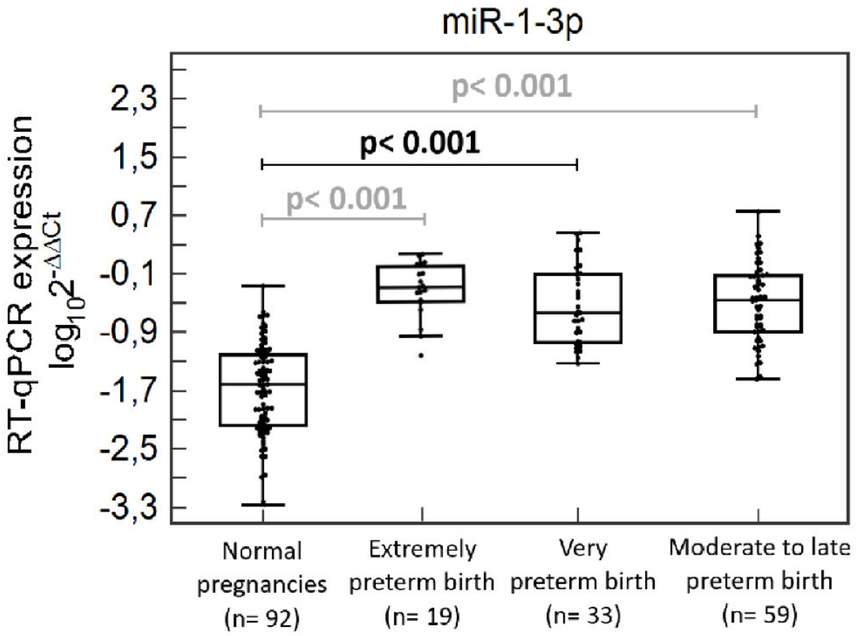

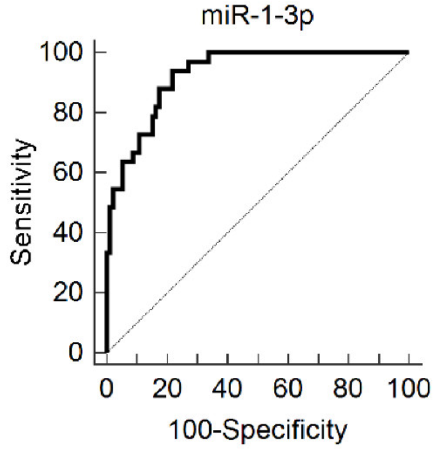

Area under the ROC curve (AUC)	0.927
Standard Error	0.0222
95% Confidence interval	0.866 to 0.966
Significance level P (Area=0.5)	<0.0001

Estimated sensitivity at fixed specificity			
Specificity	Sensitivity	95% CI	Criterion
90.00	66.67	39.39 to 81.82	>0.125703896

Criterion	Sensitivity	95% CI	Specificity	95% CI	+LR	95% CI	-LR	95% CI
>0.063365317	93.94	79.8 - 99.3	78.26	68.4 - 86.2	4.32	2.9 - 6.4	0.077	0.02 - 0.3

C

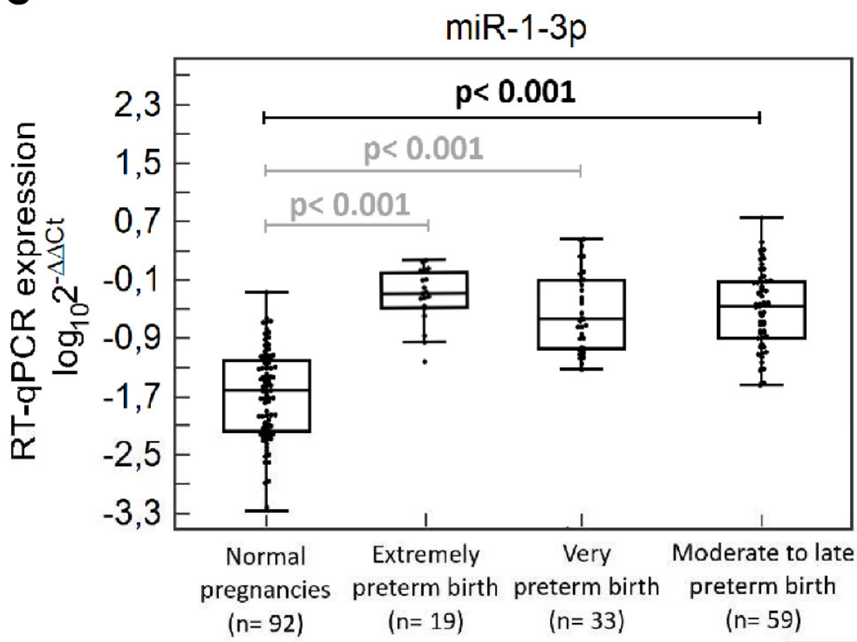

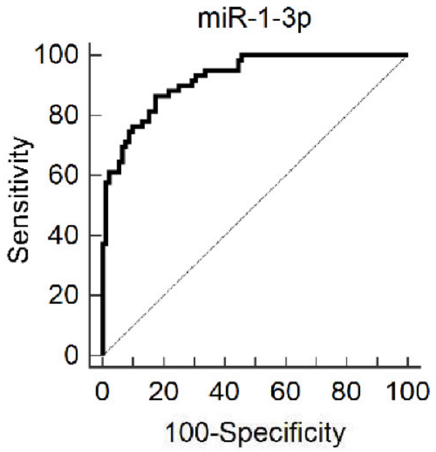

Area under the ROC curve (AUC)	0.923
Standard Error	0.0205
95% Confidence interval	0.869 to 0.960
Significance level P (Area=0.5)	<0.0001

Estimated sensitivity at fixed specificity			
Specificity	Sensitivity	95% CI	Criterion
90.00	76.27	61.02 to 89.83	>0.124151746

Criterion	Sensitivity	95% CI	Specificity	95% CI	+LR	95% CI	-LR	95% CI
>0.073354052	86.44	75.0 - 94.0	82.61	73.3 - 89.7	4.97	3.1 - 7.8	0.16	0.09 - 0.3

D

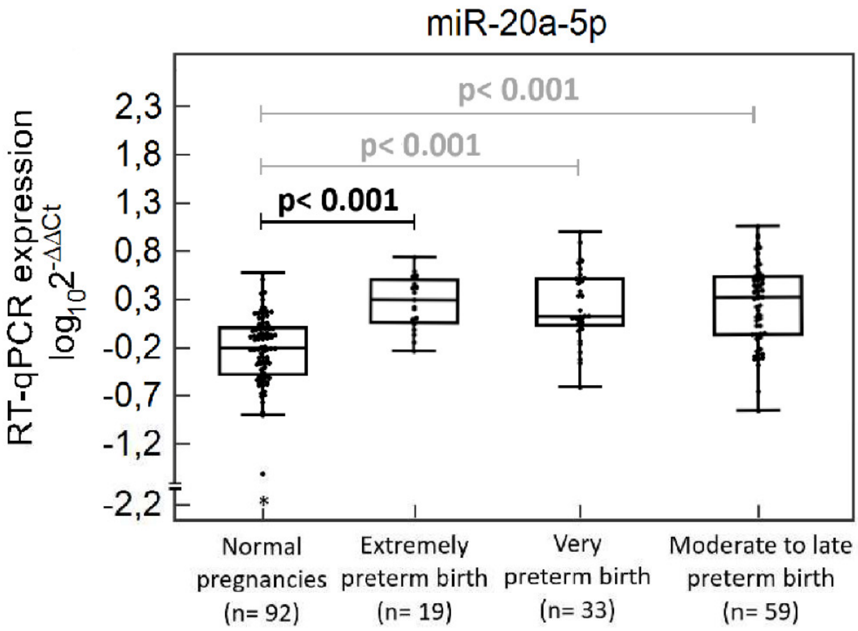

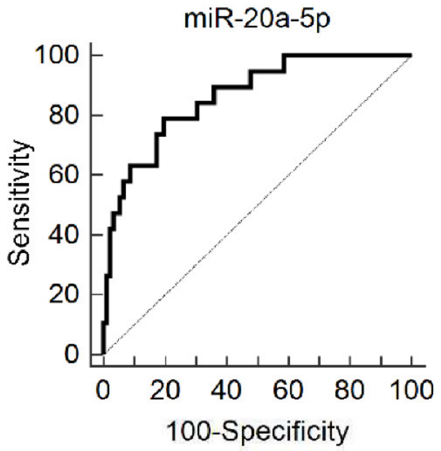

Area under the ROC curve (AUC)	0.863
Standard Error	0.0445
95% Confidence interval	0.784 to 0.921
Significance level P (Area=0.5)	<0.0001

Estimated sensitivity at fixed specificity			
Specificity	Sensitivity	95% CI	Criterion
90.00	63.16	42.11 to 84.21	>1.4726476

Criterion	Sensitivity	95% CI	Specificity	95% CI	+LR	95% CI	-LR	95% CI
>1.096585	78.95	54.4 - 93.9	80.43	70.9 - 88.0	4.04	2.5 - 6.5	0.26	0.1 - 0.6

E

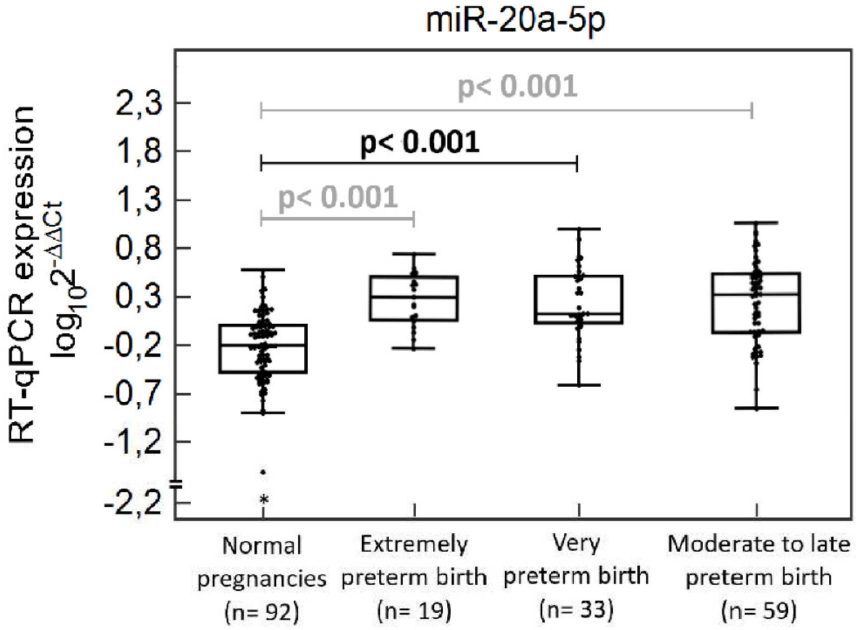

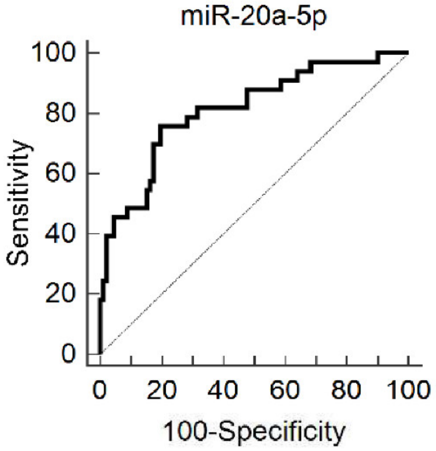

Area under the ROC curve (AUC)	0.811
Standard Error	0.0457
95% Confidence interval	0.732 to 0.876
Significance level P (Area=0.5)	<0.0001

Estimated sensitivity at fixed specificity			
Specificity	Sensitivity	95% CI	Criterion
90.00	48.48	33.33 to 69.70	>1.4726476

Criterion	Sensitivity	95% CI	Specificity	95% CI	+LR	95% CI	-LR	95% CI
>1.096585	75.76	57.7 - 88.9	80.43	70.9 - 88.0	3.87	2.5 - 6.1	0.30	0.2 - 0.6

F

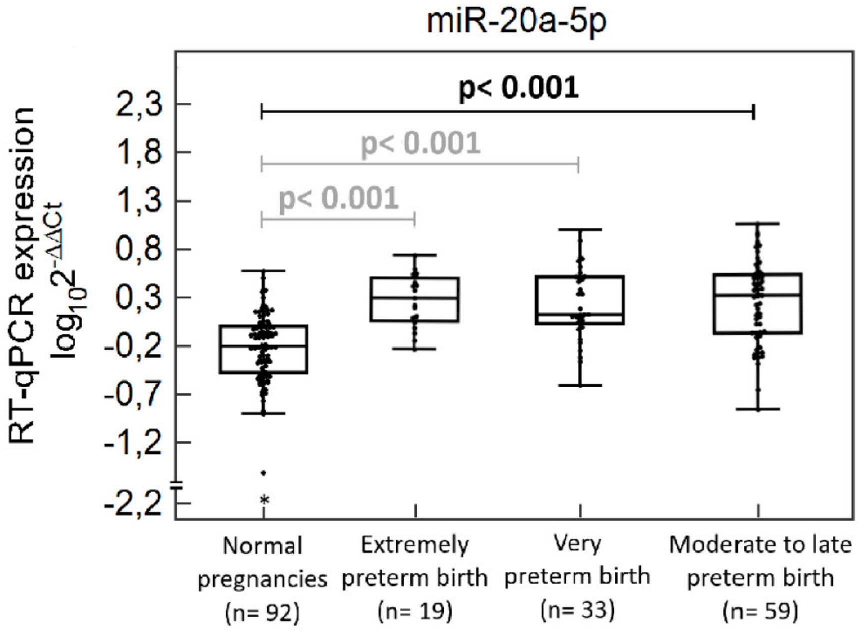

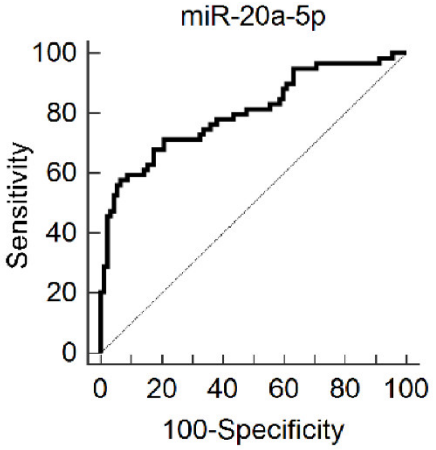

Area under the ROC curve (AUC)	0.803
Standard Error	0.0385
95% Confidence interval	0.730 to 0.863
Significance level P (Area=0.5)	<0.0001

Estimated sensitivity at fixed specificity			
Specificity	Sensitivity	95% CI	Criterion
90.00	59.32	45.76 to 72.88	>1.4726476

Criterion	Sensitivity	95% CI	Specificity	95% CI	+LR	95% CI	-LR	95% CI
>1.626209	57.63	44.1 - 70.4	93.48	86.3 - 97.6	8.84	4.0 - 19.7	0.45	0.3 - 0.6

Supplementary Figure S5. Altered Postnatal MicroRNA Expression Profile in Preterm Born Children with regard to Gestational Age at Delivery (extremely preterm, very preterm, and moderate to late preterm). Box plots and ROC curves of 2 selected individual microRNAs (miR-1-3p and miR-20a-5p) with the best sensitivity at 10.0% FPR are presented.
