# Supplementary material for: Postnatal Expression Profile of MicroRNAs Associated with Cardiovascular Diseases in 3- to 11-Year-Old Preterm-Born Children
Source: Biomedicines. 2021 Jun 24;9(7):727. doi: 10.3390/biomedicines9070727 (PMC8301298; doi:10.3390/biomedicines9070727)
Supplement: Supplementary file 1 [file biomedicines-09-00727-s001.zip › Supplementary Table S1.pdf]

Supplementary Table S1. Postnatal MicroRNA Expression Profiles in Preterm Born Children.

	**Kruskal-Wallis test results**<br>**PPROM (n=55) vs NP (n=92)**<br>**PTB (n=56) vs NP (n=92)**	**ROC curve analysis results**<br>**PPROM (n=55) vs NP (n=92)**<br>**PTB (n=56) vs NP (n=92)**
miR-1-3p	0.678±0.924 vs 0.048±0.072, **p< 0.001**	AUC 0.941, **p< 0.001**, sen 89.09%, spe 82.61%, cut off >0.0733, 78.18% at 10.0% FPR
	0.534±0.615 vs 0.048±0.072, **p< 0.001**	AUC 0.924, **p< 0.001**, sen 87.50%, spe 82.61%, cut off >0.0733, 73.21% at 10.0% FPR
miR-16-5p	1.534±1.106 vs 0.827±0.604, **p< 0.001**	AUC 0.761, **p< 0.001**, sen 63.64%, spe 82.61%, cut off >1.0999, 38.18% at 10.0% FPR
	1.729±1.595 vs 0.827±0.604, **p< 0.001**	AUC 0.799, **p< 0.001**, sen 78.57%, spe 72.83%, cut off >0.9370, 41.07% at 10.0% FPR
miR-17-5p	1.516±0.947 vs 0.972±0.766, **p< 0.001**	AUC 0.699, **p< 0.001**, sen 56.36%, spe 78.26%, cut off >1.2101, 36.36% at 10.0% FPR
	1.685±1.159 vs 0.972±0.766, **p< 0.001**	AUC 0.730, **p< 0.001**, sen 60.71%, spe 81.52%, cut off >1.2543, 42.86% at 10.0% FPR
miR-20a-5p	2.381±1.989 vs 0.800±0.630, **p< 0.001**	AUC 0.805, **p< 0.001**, sen 72.73%, spe 79.35%, cut off >1.0429, 60.00% at 10.0% FPR
	2.668±2.372 vs 0.800±0.630, **p< 0.001**	AUC 0.826, **p< 0.001**, sen 75.00%, spe 80.43%, cut off >1.0966, 53.57% at 10.0% FPR
miR-20b-5p	2.051±1.276 vs 1.126±1.050, **p< 0.001**	AUC 0.763, **p< 0.001**, sen 69.09%, spe 77.17%, cut off >1.4280, 43.64% at 10.0% FPR
	2.304±1.914 vs 1.126±1.050, **p< 0.001**	AUC 0.774, **p< 0.001**, sen 78.57%, spe 70.65%, cut off >1.1681, 50.00% at 10.0% FPR
miR-21-5p	0.427±0.535 vs 0.189±0.167, **p< 0.001**	AUC 0.738, **p< 0.001**, sen 67.27%, spe 70.65%, cut off >0.2252, 38.18% at 10.0% FPR
	0.446±0.496 vs 0.189±0.167, **p< 0.001**	AUC 0.756, **p< 0.001**, sen 85.71%, spe 56.52%, cut off >0.1672, 33.93% at 10.0% FPR

miR-23a-3p	0.201±0.198 vs 0.121±0.123, **p= 0.027**	AUC 0.628, **p= 0.008**, sen 50.91%, spe 71.74%, cut off >0.1387, 23.64% at 10.0% FPR
	0.268±0.406 vs 0.121±0.123, **p= 0.003**	AUC 0.663, **p< 0.001**, sen 73.21%, spe 55.43%, cut off >0.0849, 26.79% at 10.0% FPR
miR-24-3p	0.249±0.172 vs 0.187±0.129, **p= 0.046**	AUC 0.618, **p= 0.014**, sen 40.00%, spe 83.70%, cut off >0.2806, 18.18% at 10.0% FPR
	0.266±0.196 vs 0.187±0.129, **p= 0.010**	AUC 0.646, **p= 0.002**, sen 60.71%, spe 68.48%, cut off >0.1887, 21.43% at 10.0% FPR
miR-26a-5p	0.679±0.505 vs 0.358±0.322, **p< 0.001**	AUC 0.755, **p< 0.001**, sen 80.00%, spe 59.78%, cut off >0.3149, 40.00% at 10.0% FPR
	0.717±0.668 vs 0.358±0.322, **p< 0.001**	AUC 0.767, **p< 0.001**, sen 71.43%, spe 70.65%, cut off >0.3944, 41.07% at 10.0% FPR
miR-29a-3p	0.419±0.375 vs 0.183±0.194, **p< 0.001**	AUC 0.808, **p< 0.001**, sen 89.09%, spe 66.30%, cut off >0.1618, 34.55% at 10.0% FPR
	0.535±0.736 vs 0.183±0.194, **p< 0.001**	AUC 0.812, **p< 0.001**, sen 89.29%, spe 63.04%, cut off >0.1471, 41.07% at 10.0% FPR
miR-92a-3p	1.734±1.243 vs 1.666±1.059, p= 1.000	-
	1.920±1.556 vs 1.666±1.059, p= 1.000	-
miR-100-5p	0.003±0.003 vs 0.001±0.001, **p< 0.001**	AUC 0.729, **p< 0.001**, sen 54.55%, spe 86.96%, cut off >0.0025, 40.00% at 10.0% FPR
	0.003±0.003 vs 0.001±0.001, **p< 0.001**	AUC 0.779, **p< 0.001**, sen 83.93%, spe 60.87%, cut off >0.0013, 39.29% at 10.0% FPR
miR-103a-3p	2.195±2.132 vs 0.837±0.826, **p< 0.001**	AUC 0.768, **p< 0.001**, sen 67.27%, spe 73.91%, cut off >0.9662, 41.82% at 10.0% FPR
	2.174±1.764 vs 0.837±0.826, **p< 0.001**	AUC 0.839, **p< 0.001**, sen 83.93%, spe 73.91%, cut off >0.9662, 50.00% at 10.0% FPR
miR-125b-5p	0.007±0.006 vs 0.003±0.002, **p< 0.001**	AUC 0.735, **p< 0.001**, sen 63.64%, spe 80.43%, cut off >0.0038, 28.57% at 10.0% FPR
	0.007±0.009 vs 0.003±0.002, **p< 0.001**	AUC 0.748, **p< 0.001**, sen 69.64%, spe 76.09%, cut off >0.0034, 50.00% at 10.0% FPR

miR-126-3p	0.344±0.245 vs 0.141±0.128, **p< 0.001**	AUC 0.811, **p< 0.001**, sen 87.27%, spe 63.04%, cut off >0.1265, 47.27% at 10.0% FPR
	0.387±0.411 vs 0.141±0.128, **p< 0.001**	AUC 0.818, **p< 0.001**, sen 64.29%, spe 86.96%, cut off >0.2218, 44.64% at 10.0% FPR
miR-130b-3p	0.665±0.482 vs 0.431±0.487, **p< 0.001**	AUC 0.713, **p< 0.001**, sen 89.09%, spe 48.91%, cut off >0.2348, 16.36% at 10.0% FPR
	0.811±0.662 vs 0.431±0.487, **p< 0.001**	AUC 0.752, **p< 0.001**, sen 69.64%, spe 76.09%, cut off >0.5232, 17.86% at 10.0% FPR
miR-133a-3p	0.164±0.161 vs 0.054±0.063, **p< 0.001**	AUC 0.776, **p< 0.001**, sen 83.64%, spe 60.87%, cut off >0.0421, 38.18% at 10.0% FPR
	0.135±0.151 vs 0.054±0.063, **p< 0.001**	AUC 0.742, **p< 0.001**, sen 91.07%, spe 51.09%, cut off >0.0270, 28.57% at 10.0% FPR
miR-143-3p	0.050±0.076 vs 0.014±0.014, **p< 0.001**	AUC 0.796, **p< 0.001**, sen 69.09%, spe 79.35%, cut off >0.0184, 43.64% at 10.0% FPR
	0.047±0.046 vs 0.014±0.014, **p< 0.001**	AUC 0.860, **p< 0.001**, sen 98.21%, spe 59.78%, cut off >0.0114, 51.79% at 10.0% FPR
miR-145-5p	0.123±0.131 vs 0.063±0.039, **p= 0.005**	AUC 0.654, **p= 0.002**, sen 34.55%, spe 95.65%, cut off >0.1377, 38.18% at 10.0% FPR
	0.118±0.113 vs 0.063±0.039, **p= 0.002**	AUC 0.666, **p< 0.001**, sen 30.36%, spe 93.48%, cut off >0.1272, 30.36% at 10.0% FPR
miR-146a-5p	1.912±1.408 vs 1.022±1.054, **p< 0.001**	AUC 0.753, **p< 0.001**, sen 87.27%, spe 51.09%, cut off >0.6891, 38.18% at 10.0% FPR
	2.045±1.814 vs 1.022±1.054, **p< 0.001**	AUC 0.763, **p< 0.001**, sen 80.36%, spe 64.13%, cut off >0.9569, 33.93% at 10.0% FPR
miR-155-5p	1.871±1.852 vs 1.779±1.590, p= 1.000	-
	1.894±1.618 vs 1.779±1.590, p= 1.000	-
miR-181a-5p	0.432±0.508 vs 0.216±0.312, **p< 0.001**	AUC 0.724, **p< 0.001**, sen 54.55%, spe 82.61%, cut off >0.2972, 38.18% at 10.0% FPR
	0.414±0.292 vs 0.216±0.312, **p< 0.001**	AUC 0.795, **p< 0.001**, sen 91.07%, spe 58.70%, cut off >0.1846, 42.86% at 10.0% FPR

miR-195-5p	0.208±0.198 vs 0.069±0.109, **p< 0.001**	AUC 0.807, **p< 0.001**, sen 83.64%, spe 66.30%, cut off >0.0483, 40.00% at 10.0% FPR
	0.376±0.849 vs 0.069±0.109, **p< 0.001**	AUC 0.803, **p< 0.001**, sen 80.36%, spe 70.65%, cut off >0.0553, 41.07% at 10.0% FPR
miR-199a-5p	0.096±0.105 vs 0.048±0.090, **p< 0.001**	AUC 0.772, **p< 0.001**, sen 80.00%, spe 69.57%, cut off >0.0331, 20.00% at 10.0% FPR
	0.108±0.138 vs 0.048±0.090, **p< 0.001**	AUC 0.779, **p< 0.001**, sen 71.43%, spe 78.26%, cut off >0.0491, 25.00% at 10.0% FPR
miR-210-3p	0.088±0.079 vs 0.098±0.072, p= 0.827	-
	0.088±0.073 vs 0.098±0.072, p= 0.956	-
miR-221-3p	0.664±0.489 vs 0.302±0.263, **p< 0.001**	AUC 0.780, **p< 0.001**, sen 98.18%, spe 46.74%, cut off >0.1961, 38.18% at 10.0% FPR
	0.693±0.625 vs 0.302±0.263, **p< 0.001**	AUC 0.780, **p< 0.001**, sen 75.00%, spe 70.65%, cut off >0.3469, 32.14% at 10.0% FPR
miR-342-3p	3.470±3.301 vs 3.225±2.650, p= 1.000	-
	3.308±2.155 vs 3.225±2.650, p= 1.000	-
miR-499a-5p	0.699±1.684 vs 0.175±0.339, **p< 0.001**	AUC 0.814, **p< 0.001**, sen 69.09%, spe 81.52%, cut off >0.1899, 40.00% at 10.0% FPR
	0.798±1.311 vs 0.175±0.339, **p< 0.001**	AUC 0.843, **p< 0.001**, sen 85.71%, spe 70.65%, cut off >0.1180, 55.36% at 10.0% FPR
miR-574-3p	0.139±0.122 vs 0.075±0.051, **p< 0.001**	AUC 0.735, **p< 0.001**, sen 78.18%, spe 63.04%, cut off >0.0713, 29.09% at 10.0% FPR
	0.151±0.143 vs 0.075±0.051, **p< 0.001**	AUC 0.750, **p< 0.001**, sen 75.00%, spe 63.04%, cut off >0.0713, 26.79% at 10.0% FPR

MicroRNA gene expression is compared between individual groups using Kruskal-Wallis test. ROC curve analysis shows the potential of individual microRNAs to differentiate between children descending from normal term pregnancies and those descending from PPROM or PTB pregnancies. Statistically significant results are marked in bold. Mean

± SD values of relative fold gene expression of samples ($2^{-\Delta\Delta Ct}$) are presented. NP, normal pregnancies; PTB, spontaneous preterm birth; PPROM, preterm prelabor rupture of membranes; ROC, receiver operating characteristic; sen, sensitivity; spe, specificity; AUC, area under the ROC curve; FPR, false positive rate.
