# Supplementary material for: Postnatal Expression Profile of MicroRNAs Associated with Cardiovascular Diseases in 3- to 11-Year-Old Preterm-Born Children
Source: Biomedicines. 2021 Jun 24;9(7):727. doi: 10.3390/biomedicines9070727 (PMC8301298; doi:10.3390/biomedicines9070727)
Supplement: Supplementary file 1 [file biomedicines-09-00727-s001.zip › Supplementary Table S2.pdf]

Supplementary Table 2. Postnatal MicroRNA Expression Profiles in Children Descending from PPROM Pregnancies with regard to Clinical Findings

	**Kruskal-Wallis test results**<br>**PPROM with normal clinical findings (n=23) vs NP (n=92)**<br>**PPROM with abnormal clinical findings (n=32) vs NP (n=92)**	**ROC curve analysis results**<br>**PPROM with normal clinical findings (n=23) vs NP (n=92)**<br>**PPROM with abnormal clinical findings (n=32) vs NP (n=92)**
miR-1-3p	0.551±0.460 vs 0.033±0.040, **p< 0.001**	AUC 0.951, **p< 0.001**, sen 100.00%, spe 75.00%, cut off >0.0577, 78.26% at 10.0% FPR
	0.769±1.147 vs 0.033±0.040, **p< 0.001**	AUC 0.933, **p< 0.001**, sen 87.50%, spe 82.61%, cut off >0.0733, 78.12% at 10.0% FPR
miR-16-5p	1.260±0.654 vs 0.813±0.677, **p= 0.010**	AUC 0.739, **p< 0.001**, sen 60.87%, spe 82.61%, cut off >1.0999, 30.43% at 10.0% FPR
	1.731±1.316 vs 0.813±0.677, **p< 0.001**	AUC 0.778, **p< 0.001**, sen 62.50%, spe 86.96%, cut off >1.1942, 43.75% at 10.0% FPR
miR-17-5p	1.369±0.702 vs 0.941±0.823, **p= 0.045**	AUC 0.702, **p< 0.001**, sen 95.65%, spe 43.48%, cut off >0.6328, 30.43% at 10.0% FPR
	1.621±1.089 vs 0.941±0.823, **p= 0.005**	AUC 0.696, **p< 0.001**, sen 46.88%, spe 89.13%, cut off >1.5463, 40.63% at 10.0% FPR
miR-20a-5p	2.449±1.663 vs 0.768±0.597, **p< 0.001**	AUC 0.825, **p< 0.001**, sen 65.22%, spe 91.30%, cut off >1.4825, 65.22% at 10.0% FPR
	2.333±2.218 vs 0.768±0.597, **p< 0.001**	AUC 0.790, **p< 0.001**, sen 75.00%, spe 79.35%, cut off >1.0429, 56.25% at 10.0% FPR
miR-20b-5p	1.783±0.875 vs 1.062±0.926, **p= 0.008**	AUC 0.760, **p< 0.001**, sen 91.30%, spe 59.78%, cut off >0.9535, 34.78% at 10.0% FPR
	2.243±1.484 vs 1.062±0.926, **p< 0.001**	AUC 0.765, **p< 0.001**, sen 71.87%, spe 77.17%, cut off >1.4280, 50.00% at 10.0% FPR
miR-21-5p	0.313±0.204 vs 0.156±0.127, **p= 0.009**	AUC 0.702, **p< 0.001**, sen 52.17%, spe 85.87%, cut off >0.3232, 34.78% at 10.0% FPR
	0.509±0.673 vs 0.156±0.127, **p< 0.001**	AUC 0.764, **p< 0.001**, sen 84.37%, spe 63.04%, cut off >0.1990, 40.63% at 10.0% FPR
miR-23a-3p	0.161±0.160 vs 0.115±0.130, p= 1.000	-
	0.229±0.219 vs 0.115±0.130, **p= 0.003**	AUC 0.682, **p< 0.001**, sen 75.00%, spe 57.61%, cut off >0.0955, 28.12% at 10.0% FPR

miR-24-3p	0.225±0.117 vs 0.184±0.136, p= 1.000	-
	0.267±0.202 vs 0.184±0.136, p= 0.343	AUC 0.624, **p= 0.039**, sen 50.00%, spe 78.26%, cut off >0.2641, 21.87% at 10.0% FPR
miR-26a-5p	0.618±0.380 vs 0.363±0.376, **p= 0.009**	AUC 0.735, **p< 0.001**, sen 60.87%, spe 78.26%, cut off >0.4833, 34.78% at 10.0% FPR
	0.722±0.581 vs 0.363±0.376, **p< 0.001**	AUC 0.769, **p< 0.001**, sen 84.37%, spe 59.78%, cut off >0.3149, 43.75% at 10.0% FPR
miR-29a-3p	0.375±0.202 vs 0.168±0.172, **p< 0.001**	AUC 0.802, **p< 0.001**, sen 82.61%, spe 71.74%, cut off >0.1840, 39.13% at 10.0% FPR
	0.451±0.462 vs 0.168±0.172, **p< 0.001**	AUC 0.813, **p< 0.001**, sen 90.62%, spe 66.30%, cut off >0.1618, 31.25% at 10.0% FPR
miR-92a-3p	1.448±0.926 vs 1.664±1.090, p= 1.000	-
	1.940±1.407 vs 1.664±1.090, p= 1.000	-
miR-100-5p	0.003±0.003 vs 0.001±0.001, **p= 0.001**	AUC 0.744, **p< 0.001**, sen 56.52%, spe 86.96%, cut off >0.0025, 43.48% at 10.0% FPR
	0.003±0.003 vs 0.001±0.001, **p< 0.001**	AUC 0.717, **p< 0.001**, sen 62.50%, spe 79.35%, cut off >0.0019, 37.50% at 10.0% FPR
miR-103a-3p	1.780±2.054 vs 0.818±0.924, **p= 0.033**	AUC 0.715, **p< 0.001**, sen 65.22%, spe 67.39%, cut off >0.8740, 30.43% at 10.0% FPR
	2.493±2.169 vs 0.818±0.924, **p< 0.001**	AUC 0.806, **p< 0.001**, sen 75.00%, spe 75.00%, cut off >1.0081, 50.00% at 10.0% FPR
miR-125b-5p	0.007±0.006 vs 0.003±0.002, **p= 0.009**	AUC 0.722, **p< 0.001**, sen 60.87%, spe 82.61%, cut off >0.0038, 39.13% at 10.0% FPR
	0.006±0.006 vs 0.003±0.002, **p< 0.001**	AUC 0.744, **p< 0.001**, sen 68.75%, spe 78.26%, cut off >0.0035, 31.25% at 10.0% FPR
miR-126-3p	0.304±0.183 vs 0.123±0.098, **p< 0.001**	AUC 0.813, **p< 0.001**, sen 86.96%, spe 63.04%, cut off >0.1265, 52.17% at 10.0% FPR
	0.372±0.281 vs 0.123±0.098, **p< 0.001**	AUC 0.809, **p< 0.001**, sen 87.50%, spe 63.04%, cut off >0.1265, 43.75% at 10.0% FPR
miR-130b-3p	0.617±0.435 vs 0.489±0.595, p= 0.256	AUC 0.701, **p< 0.001**, sen 86.96%, spe 51.09%, cut off >0.2514, 17.39% at 10.0% FPR
	0.700±0.517 vs 0.489±0.595, **p= 0.016**	AUC 0.722, **p< 0.001**, sen 90.62%, spe 48.91%, cut off >0.2348, 15.63% at 10.0% FPR

miR-133a-3p	0.146±0.133 vs 0.040±0.037, **p< 0.001**	AUC 0.801, **p< 0.001**, sen 91.30%, spe 60.87%, cut off >0.0421, 34.78% at 10.0% FPR
	0.177±0.179 vs 0.040±0.037, **p< 0.001**	AUC 0.758, **p< 0.001**, sen 71.87%, spe 68.48%, cut off >0.0557, 40.63% at 10.0% FPR
miR-143-3p	0.033±0.029 vs 0.014±0.014, **p= 0.007**	AUC 0.749, **p< 0.001**, sen 82.61%, spe 56.52%, cut off >0.0105, 34.78% at 10.0% FPR
	0.063±0.095 vs 0.014±0.014, **p< 0.001**	AUC 0.829, **p< 0.001**, sen 78.12%, spe 79.35%, cut off >0.0184, 50.00% at 10.0% FPR
miR-145-5p	0.085±0.063 vs 0.061±0.036, p= 1.000	-
	0.149±0.159 vs 0.061±0.036, **p= 0.012**	AUC 0.702, **p< 0.001**, sen 43.75%, spe 95.65%, cut off >0.1377, 46.88% at 10.0% FPR
miR-146a-5p	1.788±0.815 vs 0.955±1.056, **p< 0.001**	AUC 0.791, **p< 0.001**, sen 100.00%, spe 46.74%, cut off >0.6136, 47.83% at 10.0% FPR
	2.002±1.720 vs 0.955±1.056, **p= 0.001**	AUC 0.725, **p< 0.001**, sen 84.37%, spe 51.09%, cut off >0.6891, 31.25% at 10.0% FPR
miR-155-5p	1.700±1.443 vs 1.628±1.197, p= 1.000	-
	1.993±1.044 vs 1.623±1.197, p= 0.975	-
miR-181a-5p	0.339±0.240 vs 0.175±0.116, p= 0.053	AUC 0.695, **p= 0.002**, sen 78.26%, spe 57.61%, cut off >0.1805, 26.09% at 10.0% FPR
	0.498±0.631 vs 0.175±0.116, **p< 0.001**	AUC 0.745, **p< 0.001**, sen 62.50%, spe 82.61%, cut off >0.2972, 46.88% at 10.0% FPR
miR-195-5p	0.202±0.199 vs 0.073±0.116, **p< 0.001**	AUC 0.806, **p< 0.001**, sen 95.65%, spe 56.52%, cut off >0.0319, 39.13% at 10.0% FPR
	0.212±0.200 vs 0.073±0.116, **p< 0.001**	AUC 0.807, **p< 0.001**, sen 81.25%, spe 70.65%, cut off >0.0553, 40.63% at 10.0% FPR
miR-199a-5p	0.086±0.078 vs 0.059±0.111, **p= 0.029**	AUC 0.749, **p< 0.001**, sen 73.91%, spe 69.57%, cut off >0.0331, 17.39% at 10.0% FPR
	0.103±0.122 vs 0.059±0.111, **p< 0.001**	AUC 0.789, **p< 0.001**, sen 78.12%, spe 76.09%, cut off >0.0417, 21.87% at 10.0% FPR
miR-210-3p	0.079±0.051 vs 0.095±0.072, p= 1.000	-
	0.094±0.094 vs 0.095±0.072, p= 1.000	-

miR-221-3p	0.558±0.340 vs 0.276±0.229, **p= 0.003**	AUC 0.758, **p< 0.001**, sen 100.00%, spe 46.74%, cut off >0.1961, 30.43% at 10.0% FPR
	0.740±0.567 vs 0.276±0.229, **p< 0.001**	AUC 0.796, **p< 0.001**, sen 75.00%, spe 70.65%, cut off >0.3469, 43.75% at 10.0% FPR
miR-342-3p	2.972±1.720 vs 3.052±1.817, p= 1.000	-
	3.828±4.070 vs 3.052±1.817, p= 1.000	-
miR-499a-5p	0.446±0.418 vs 0.147±0.243, **p= 0.001**	AUC 0.784, **p< 0.001**, sen 82.61%, spe 64.13%, cut off >0.1011, 39.13% at 10.0% FPR
	0.882±2.176 vs 0.147±0.243, **p< 0.001**	AUC 0.835, **p< 0.001**, sen 87.50%, spe 71.74%, cut off >0.1188, 40.63% at 10.0% FPR
miR-574-3p	0.106±0.055 vs 0.074±0.052, p= 0.097	AUC 0.692, **p= 0.002**, sen 82.61%, spe 63.04%, cut off >0.0713, 21.74% at 10.0% FPR
	0.162±0.149 vs 0.074±0.052, **p< 0.001**	AUC 0.766, **p< 0.001**, sen 68.75%, spe 70.65%, cut off >0.0832, 34.38% at 10.0% FPR

MicroRNA gene expression is compared between individual groups using Kruskal-Wallis test. ROC curve analysis shows the potential of individual microRNAs to differentiate between children descending from normal term pregnancies and those descending from PPROM pregnancies with normal or abnormal clinical findings. Statistically significant results are marked in bold. Mean ± SD values of relative fold gene expression of samples ($2^{-\Delta\Delta Ct}$) are presented. NP, normal pregnancies; PPROM, preterm prelabor rupture of membranes; ROC, receiver operating characteristic; AUC, area under the ROC curve; sen, sensitivity; spe, specificity; FPR, false positive rate.
