# Supplementary material for: Postnatal Expression Profile of MicroRNAs Associated with Cardiovascular Diseases in 3- to 11-Year-Old Preterm-Born Children
Source: Biomedicines. 2021 Jun 24;9(7):727. doi: 10.3390/biomedicines9070727 (PMC8301298; doi:10.3390/biomedicines9070727)
Supplement: Supplementary file 1 [file biomedicines-09-00727-s001.zip › Supplementary Table S3.pdf]

Supplementary Table S3. Postnatal MicroRNA Expression Profiles in Children Descending from PTB Pregnancies with regard to Clinical Findings.

	**Kruskal-Wallis test results**<br>**PTB with normal clinical findings (n=22) vs NP (n=92)**<br>**PTB with abnormal clinical findings (n=34) vs NP (n=92)**	**ROC curve analysis results**<br>**PTB with normal clinical findings (n=22) vs NP (n=92)**<br>**PTB with abnormal clinical findings (n=34) vs NP (n=92)**
miR-1-3p	0.459±0.546 vs 0.033±0.040, **p< 0.001**	AUC 0.896, **p< 0.001**, sen 81.82%, spe 84.78%, cut off >0.0885, 68.18% at 10.0% FPR
	0.583±0.659 vs 0.033±0.040, **p< 0.001**	AUC 0.942, **p< 0.001**, sen 91.18%, spe 82.61%, cut off >0.0733, 76.47% at 10.0% FPR
miR-16-5p	1.776±1.954 vs 0.813±0.677, **p= 0.001**	AUC 0.781, **p< 0.001**, sen 81.82%, spe 66.30%, cut off >0.8271, 31.82% at 10.0% FPR
	1.698±1.345 vs 0.813±0.677, **p< 0.001**	AUC 0.811, **p< 0.001**, sen 82.35%, spe 72.83%, cut off >0.9370, 47.06% at 10.0% FPR
miR-17-5p	1.580±1.149 vs 0.941±0.823, p= 0.058	AUC 0.686, **p= 0.006**, sen 50.00%, spe 89.13%, cut off >1.5463, 40.91% at 10.0% FPR
	1.752±1.178 vs 0.941±0.823, **p= 0.005**	AUC 0.758, **p< 0.001**, sen 73.53%, spe 72.83%, cut off >1.0995, 44.12% at 10.0% FPR
miR-20a-5p	2.181±2.147 vs 0.768±0.597, **p< 0.001**	AUC 0.812, **p< 0.001**, sen 72.73%, spe 80.43%, cut off >1.0966, 50.00% at 10.0% FPR
	2.984±2.487 vs 0.768±0.597, **p< 0.001**	AUC 0.835, **p< 0.001**, sen 76.47%, spe 80.43%, cut off >1.0966, 55.88% at 10.0% FPR
miR-20b-5p	2.256±1.990 vs 1.062±0.926, **p= 0.019**	AUC 0.716, **p< 0.001**, sen 68.18%, spe 73.91%, cut off >1.3508, 36.36% at 10.0% FPR
	2.335±1.893 vs 1.062±0.926, **p< 0.001**	AUC 0.811, **p< 0.001**, sen 85.29%, spe 70.65%, cut off >1.1681, 58.82% at 10.0% FPR
miR-21-5p	0.447±0.433 vs 0.156±0.127, **p< 0.001**	AUC 0.782, **p< 0.001**, sen 68.18%, spe 77.17%, cut off >0.2485, 40.91% at 10.0% FPR
	0.446±0.539 vs 0.156±0.127, **p< 0.001**	AUC 0.739, **p< 0.001**, sen 85.29%, spe 56.52%, cut off >0.1672, 29.41% at 10.0% FPR
miR-23a-3p	0.274±0.392 vs 0.115±0.130, **p= 0.028**	AUC 0.707, **p< 0.001**, sen 81.82%, spe 55.43%, cut off >0.0849, 22.73% at 10.0% FPR
	0.264±0.421 vs 0.115±0.130, p= 0.134	AUC 0.635, **p= 0.022**, sen 41.18%, spe 84.78%, cut off >0.2119, 29.41% at 10.0% FPR

miR-24-3p	0.250±0.159 vs 0.184±0.136, p= 0.786	-
	0.277±0.218 vs 0.184±0.136, p= 0.087	AUC 0.658, **p= 0.004**, sen 73.53%, spe 58.70%, cut off >0.2119, 23.53% at 10.00 FPR
miR-26a-5p	0.628±0.405 vs 0.363±0.376, **p= 0.013**	AUC 0.729, **p< 0.001**, sen 50.00%, spe 86.96%, cut off >0.5945, 40.91% at 10.0% FPR
	0.774±0.794 vs 0.363±0.376, **p< 0.001**	AUC 0.792, **p< 0.001**, sen 88.24%, spe 59.78%, cut off >0.3149, 41.18% at 10.0% FPR
miR-29a-3p	0.417±0.323 vs 0.168±0.172, **p< 0.001**	AUC 0.797, **p< 0.001**, sen 86.36%, spe 63.04%, cut off >0.1471, 54.45% at 10.0% FPR
	0.611±0.906 vs 0.168±0.172, **p< 0.001**	AUC 0.822, **p< 0.001**, sen 85.29%, spe 71.74%, cut off >0.1840, 38.24% at 10.0% FPR
miR-92a-3p	1.836±1.809 vs 1.664±1.090, p= 1.000	-
	1.975±1.395 vs 1.664±1.090, p= 1.000	-
miR-100-5p	0.003±0.003 vs 0.001±0.001, **p= 0.006**	AUC 0.734, **p< 0.001**, sen 77.27%, spe 60.87%, cut off >0.0013, 36.36% at 10.0% FPR
	0.003±0.003 vs 0.001±0.001, **p< 0.001**	AUC 0.809, **p< 0.001**, sen 94.12%, spe 55.43%, cut off >0.0012, 41.189% at 10.0% FPR
miR-103a-3p	2.103±1.127 vs 0.818±0.924, **p< 0.001**	AUC 0.851, **p< 0.001**, sen 90.91%, spe 66.30%, cut off >0.8274, 54.55% at 10.0% FPR
	2.220±2.091 vs 0.818±0.924, **p< 0.001**	AUC 0.830, **p< 0.001**, sen 88.24%, spe 73.91%, cut off >0.9662, 47.06% at 10.0% FPR
miR-125b-5p	0.007±0.009 vs 0.003±0.002, **p= 0.048**	AUC 0.713, **p= 0.001**, sen 77.27%, spe 65.22%, cut off >0.0028, 22.73% at 10.0% FPR
	0.007±0.009 vs 0.003±0.002, **p< 0.001**	AUC 0.771, **p< 0.001**, sen 73.53%, spe 77.17%, cut off >0.0034, 32.35% at 10.0% FPR
miR-126-3p	0.336±0.333 vs 0.123±0.098, **p< 0.001**	AUC 0.793, **p< 0.001**, sen 86.36%, spe 63.04%, cut off >0.1265, 36.36% at 10.0% FPR
	0.420±0.456 vs 0.123±0.098, **p< 0.001**	AUC 0.835, **p< 0.001**, sen 79.41%, spe 79.35%, cut off >0.2053, 50.00% at 10.0% FPR
miR-130b-3p	0.681±0.569 vs 0.489±0.595, p= 0.230	AUC 0.681, **p= 0.002**, sen 81.82%, spe 51.09%, cut off >0.2514, 13.64% at 10.0% FPR
	0.895±0.711 vs 0.489±0.595, **p< 0.001**	AUC 0.798, **p< 0.001**, sen 82.35%, spe 76.09%, cut off >0.5232, 20.59% at 10.0% FPR

miR-133a-3p	0.112±0.149 vs 0.040±0.037, p= 0.066	AUC 0.685, **p= 0.002**, sen 81.82%, spe 51.09%, cut off >0.0270, 22.73% at 10.0% FPR
	0.150±0.153 vs 0.040±0.037, **p< 0.001**	AUC 0.778, **p< 0.001**, sen 97.06%, spe 51.09%, cut off >0.0270, 32.35% at 10.0% FPR
miR-143-3p	0.050±0.034 vs 0.014±0.014, **p< 0.001**	AUC 0.883, **p< 0.001**, sen 100.00%, spe 60.87%, cut off >0.0114, 54.55% at 10.0% FPR
	0.045±0.053 vs 0.014±0.014, **p< 0.001**	AUC 0.845, **p< 0.001**, sen 94.12%, spe 64.13%, cut off >0.0125, 50.00% at 10.0% FPR
miR-145-5p	0.127±0.101 vs 0.061±0.036, p= 0.126	AUC 0.681, **p= 0.014**, sen 40.91%, spe 98.91%, cut off >0.1544, 40.91% at 10.0% FPR
	0.111±0.121 vs 0.061±0.036, p= 0.181	AUC 0.656, **p= 0.003**, sen 79.41%, spe 47.83%, cut off >0.0552, 23.53% at 10.0% FPR
miR-146a-5p	2.056±2.098 vs 0.955±1.056, **p= 0.012**	AUC 0.723, **p< 0.001**, sen 95.45%, spe 43.48%, cut off >0.5950, 27.27% at 10.0% FPR
	2.038±1.639 vs 0.955±1.056, **p< 0.001**	AUC 0.789, **p< 0.001**, sen 88.24%, spe 64.13%, cut off >0.9569, 38.24% at 10.0% FPR
miR-155-5p	1.828±1.650 vs 1.623±1.197, p= 0.989	-
	1.936±1.603 vs 1.623±1.197, p= 0.965	-
miR-181a-5p	0.391±0.245 vs 0.175±0.116, **p= 0.002**	AUC 0.769, **p< 0.001**, sen 90.91%, spe 58.70%, cut off >0,1846, 40.91% at 10.0% FPR
	0.429±0.322 vs 0.175±0.116, **p< 0.001**	AUC 0.812, **p< 0.001**, sen 85.29%, spe 68.48%, cut off >0.2152, 44.12% at 10.0% FPR
miR-195-5p	0.183±0.173 vs 0.073±0.116, **p= 0.006**	AUC 0.757, **p< 0.001**, sen 72.73%, spe 71.74%, cut off >0.0586, 36.36% at 10.0% FPR
	0.501±1.069 vs 0.073±0.116, **p< 0.001**	AUC 0.833, **p< 0.001**, sen 85.29%, spe 70.65%, cut off >0.0553, 44.12% at 10.0% FPR
miR-199a-5p	0.090±0.073 vs 0.059±0.111, **p= 0.009**	AUC 0.768, **p< 0.001**, sen 86.36%, spe 63.30%, cut off >0.0324, 27.27% at 10.0% FPR
	0.119±0.167 vs 0.059±0.111, **p< 0.001**	AUC 0.786, **p< 0.001**, sen 79.41%, spe 72.83%, cut off >0.0388, 23.53% at 10.0% FPR
miR-210-3p	0.092±0.080 vs 0.095±0.072, p= 1.000	-
	0.086±0.069 vs 0.095±0.072, p= 1.000	-

miR-221-3p	0.761±0.786 vs 0.276±0.229, **p= 0.002**	AUC 0.767, **p< 0.001**, sen 72.73%, spe 70.65%, cut off >0.3469, 31.82% at 10.0% FPR
	0.650±0.503 vs 0.276±0.229, **p< 0.001**	AUC 0.789, **p< 0.001**, sen 73.53%, spe 76.09%, cut off >0.4009, 32.35% at 10.0% FPR
miR-342-3p	3.112±1.911 vs 3.052±1.817, p= 1.000	-
	3.435±2.318 vs 3.052±1.817, p= 1.000	-
miR-499a-5p	0.833±0.803 vs 0.147±0.243, **p< 0.001**	AUC 0.884, **p< 0.001**, sen 77.27%, spe 90.22%, cut off >0.3725, 77.27% at 10.0% FPR
	0.775±1.566 vs 0.147±0.243, **p< 0.001**	AUC 0.816, **p< 0.001**, sen 82.35%, spe 70.65%, cut off >0.1180, 55.36% at 10.0% FPR
miR-574-3p	0.153±0.117 vs 0.074±0.052, **p= 0.001**	AUC 0.773, **p< 0.001**, sen 75.00%, spe 63.04%, cut off >0.0878, 31.82% at 10.0% FPR
	0.150±0.160 vs 0.074±0.052, **p= 0.002**	AUC 0.735, **p< 0.001**, sen 73.53%, spe 64.13%, cut off >0.0738, 23.53% at 10.0% FPR

MicroRNA gene expression is compared between individual groups using Kruskal-Wallis test. ROC curve analysis shows the potential of individual microRNAs to differentiate between children descending from normal term pregnancies and those descending from PTB pregnancies with normal or abnormal clinical findings. Statistically significant results are marked in bold. Mean ± SD values of relative fold gene expression of samples ($2^{-\Delta\Delta Ct}$) are presented. NP, normal pregnancies; PTB, spontaneous preterm birth; ROC, receiver operating characteristic; AUC, area under the ROC curve; sen, sensitivity; spe, specificity; FPR, false positive rate.
