# Supplementary material for: Postnatal Expression Profile of MicroRNAs Associated with Cardiovascular Diseases in 3- to 11-Year-Old Preterm-Born Children
Source: Biomedicines. 2021 Jun 24;9(7):727. doi: 10.3390/biomedicines9070727 (PMC8301298; doi:10.3390/biomedicines9070727)
Supplement: Supplementary file 1 [file biomedicines-09-00727-s001.zip › Supplementary Table S4.pdf]

Supplementary Table S4. Postnatal MicroRNA Expression Profiles in Children Descending from Preterm Pregnancies with regard to Gestational Age at Delivery.

	**Extremely preterm (n=19) vs NP (n= 92)**	**Very preterm (n= 33) vs NP (n=92)**	**Moderate to late preterm (n= 59) vs NP (n=92)**
miR-1-3p	0.602±0.405 vs 0.048±0.072, **p< 0.001**<br>AUC 0.970**, p< 0.001**, sen 84.21%, spe 98.91%,<br>cut off >0.2300, 89.47% at 10.0% FPR	0.593±0.774 vs 0.048±0.072, **p< 0.001**<br>AUC 0.927**, p< 0.001**, sen 93.94%, spe 78.26%,<br>cut off >0.0634, 66.67% at 10.0% FPR	0.613±0.884 vs 0.048±0.072, **p< 0.001**<br>AUC 0.923**, p< 0.001**, sen 86.44%, spe 82.61%,<br>cut off >0.0733, 76.27% at 10.0% FPR
miR-16-5p	1.492±1.453 vs 0.827±0.604, **p= 0.007**<br>AUC 0.755**, p< 0.001**, sen 94.74%, spe 50.00%,<br>cut off >0.6757, 26.32% at 10.0% FPR	1.737±1.581 vs 0.827±0.604, **p< 0.001**<br>AUC 0.819**, p< 0.001**, sen 84.85%, spe 72.83%,<br>cut off >0.9370, 36.36% at 10.0% FPR	1.619±1.234 vs 0.827±0.604, **p< 0.001**<br>AUC 0.767**, p< 0.001**, sen 76.27%, spe 67.39%,<br>cut off >0.8467, 45.76% at 10.0% FPR
miR-17-5p	1.563±0.775 vs 0.972±0.766, **p= 0.006**<br>AUC 0.753**, p< 0.001**, sen 68.42%, spe 78.26%,<br>cut off >1.2101, 42.11% at 10.0% FPR	1.582±1.143 vs 0.972±0.766, **p= 0.007**<br>AUC 0.685**, p= 0.001**, sen 60.61%, spe 72.83%,<br>cut off >1.0995, 36.36% at 10.0% FPR	1.624±1.102 vs 0.972±0.766, **p< 0.001**<br>AUC 0.719**, p< 0.001**, sen 59.32%, spe 81.52%,<br>cut off >1.2543, 40.68% at 10.0% FPR
miR-20a-5p	2.190±1.287 vs 0.800±0.630, **p< 0.001**<br>AUC 0.863**, p< 0.001**, sen 78.95%, spe 80.43%,<br>cut off >1.0966, 63.16% at 10.0% FPR	2.413±2.134 vs 0.800±0.630, **p< 0.001**<br>AUC 0.811**, p< 0.001**, sen 75.76%, spe 80.43%,<br>cut off >1.0966, 48.48% at 10.0% FPR	2.697±2.440 vs 0.800±0.630, **p< 0.001**<br>AUC 0.803**, p< 0.001**, sen 57.63%, spe 93.48%,<br>cut off >1.6262, 59.32% at 10.0% FPR
miR-20b-5p	1.845±1.171 vs 1.126±1.050, **p= 0.008**<br>AUC 0.755**, p< 0.001**, sen 84.21%, spe 66.30%,<br>cut off >1.1001, 36.84% at 10.0% FPR	2.328±1.614 vs 1.126±1.050, **p< 0.001**<br>AUC 0.796**, p< 0.001**, sen 84.85%, spe 70.65%,<br>cut off >1.1681, 51.52% at 10.0% FPR	2.203±1.763 vs 1.126±1.050, **p< 0.001**<br>AUC 0.757**, p< 0.001**, sen 67.80%, spe 77.17%,<br>cut off >1.4280, 47.46% at 10.0% FPR
miR-21-5p	0.471±0.827 vs 0.189±0.167, **p= 0.027**	0.377±0.263 vs 0.189±0.167, **p< 0.001**	0.459±0.497 vs 0.189±0.167, **p< 0.001**

	AUC 0.725**, p< 0.001**, sen 78.95%, spe 61.96%, cut off >0.1876, 21.05% at 10.0% FPR	AUC 0.766**, p< 0.001**, sen 78.79%, spe 63.04%, cut off >0.1990, 33.33% at 10.0% FPR	AUC 0.743**, p< 0.001**, sen 59.32%, spe 78.26%, cut off >0.2544, 42.37% at 10.0% FPR
miR-23a-3p	0.252±0.276 vs 0.121±0.123, p= 0.163<br>AUC 0.658**, p= 0.031**, sen 52.63%, spe 75.00%, cut off >0.1573, 31.58% at 10.0% FPR	0.208±0.332 vs 0.121±0.123, p= 0.536<br>-	0.244±0.332 vs 0.121±0.123, **p= 0.003**<br>AUC 0.665**, p< 0.001**, sen 57.63%, spe 71.74%, cut off >0.1387, 28.81% at 10.0% FPR
miR-24-3p	0.255±0.221 vs 0.187±0.129, p= 0.806<br>-	0.238±0.144 vs 0.187±0.129, p= 0.353<br>-	0.270±0.193 vs 0.187±0.129, **p= 0.010**<br>AUC 0.650**, p= 0.001**, sen 42.37%, spe 83.70%, cut off >0.2806, 22.03% at 10.0% FPR
miR-26a-5p	0.664±0.654 vs 0.358±0.322, **p= 0.013**<br>AUC 0.742**, p< 0.001**, sen 94.74%, spe 47.83%, cut off >0.2564, 31.58% at 10.0% FPR	0.661±0.375 vs 0.358±0.322, **p< 0.001**<br>AUC 0.785**, p< 0.001**, sen 66.67%, spe 81.52%, cut off >0.5119, 36.36% at 10.0% FPR	0.729±0.670 vs 0.358±0.322, **p< 0.001**<br>AUC 0.754**, p< 0.001**, sen 50.85%, spe 88.04%, cut off >0.6078, 45.76% at 10.0% FPR
miR-29a-3p	0.457±0.577 vs 0.183±0.194, **p= 0.001**<br>AUC 0.793**, p< 0.001**, sen 84.21%, spe 71.74%, cut off >0.1840, 31.58% at 10.0% FPR	0.455±0.321 vs 0.183±0.194, **p< 0.001**<br>AUC 0.833**, p< 0.001**, sen 81.82%, spe 75.00%, cut off >0.2279, 42.42% at 10.0% FPR	0.496±0.700 vs 0.183±0.194, **p< 0.001**<br>AUC 0.803**, p< 0.001**, sen 89.83%, spe 63.04%, cut off >0.1471, 37.29% at 10.0% FPR
miR-92a-3p	1.728±1.523 vs 1.666±1.059, p= 1.000<br>-	2.043±1.472 vs 1.666±1.059, p= 1.000<br>-	1.740±1.341 vs 1.666±1.059, p= 1.000<br>-
miR-100-5p	0.003±0.003 vs 0.001±0.001, **p= 0.032**<br>AUC 0.711**, p< 0.001**, sen 84.21%, spe 55.43%,	0.003±0.003 vs 0.001±0.001, **p< 0.001**<br>AUC 0.788**, p< 0.001**, sen 81.82%, spe 71.74%,	0.003±0.003 vs 0.001±0.001, **p< 0.001**<br>AUC 0.749**, p< 0.001**, sen 55.93%, spe 86.96%,

	cut off >0.0012, 26.32% at 10.0% FPR	cut off >0.0015, 42.42% at 10.0% FPR	cut off >0.0025, 42.37% at 10.0% FPR
miR-103a-3p	2.249±2.144 vs 0.837±0.826, **p< 0.001**<br>AUC 0.811**, p< 0.001**, sen 68.42%, spe 79.35%,<br>cut off >1.2544, 47.37% at 10.0% FPR	2.070±1.496 vs 0.837±0.826, **p< 0.001**<br>AUC 0.805**, p< 0.001**, sen 78.79%, spe 73.91%,<br>cut off >0.9662, 42.42% at 10.0% FPR	2.229±2.124 vs 0.837±0.826, **p< 0.001**<br>AUC 0.800**, p< 0.001**, sen 74.58%, spe 75.00%,<br>cut off >1.0081, 47.46% at 10.0% FPR
miR-125b-5p	0.006±0.005 vs 0.003±0.002, **p= 0.017**<br>AUC 0.718**, p= 0.002**, sen 63.16%, spe 77.17%,<br>cut off >0.0034, 31.58% at 10.0% FPR	0.007±0.008 vs 0.003±0.002, **p< 0.001**<br>AUC 0.749**, p< 0.001**, sen 72.73%, spe 77.17%,<br>cut off >0.0034, 33.33% at 10.0% FPR	0.007±0.008 vs 0.003±0.002, **p< 0.001**<br>AUC 0.745**, p< 0.001**, sen 64.41%, spe 78.26%,<br>cut off >0.0035, 30.51% at 10.0% FPR
miR-126-3p	0.296±0.186 vs 0.141±0.128, **p< 0.001**<br>AUC 0.813**, p< 0.001**, sen 94.74%, spe 55.43%,<br>cut off >0.1151, 42.11% at 10.0% FPR	0.361±0.266 vs 0.141±0.128, **p< 0.001**<br>AUC 0.831**, p< 0.001**, sen 87.88%, spe 69.57%,<br>cut off >0.1503, 39.39% at 10.0% FPR	0.390±0.406 vs 0.141±0.128, **p< 0.001**<br>AUC 0.806**, p< 0.001**, sen 84.75%, spe 63.04%,<br>cut off >0.1265, 50.85% at 10.0% FPR
miR-130b-3p	0.757±0.650 vs 0.431±0.487, **p= 0.020**<br>AUC 0.714**, p< 0.001**, sen 84.21%, spe 53.26%,<br>cut off >0.2890, 15.79% at 10.0% FPR	0.680±0.425 vs 0.431±0.487, **p< 0.001**<br>AUC 0.728**, p< 0.001**, sen 66.67%, spe 79.35%,<br>cut off >0.5528, 18.18% at 10.0% FPR	0.766±0.639 vs 0.431±0.487, **p< 0.001**<br>AUC 0.742**, p< 0.001**, sen 91.53%, spe 51.09%,<br>cut off >0.2514, 16.95% at 10.0% FPR
miR-133a-3p	0.140±0.111 vs 0.054±0.063, **p< 0.001**<br>AUC 0.824**, p< 0.001**, sen 78.95%, spe 75.00%,<br>cut off >0.0678, 42.11% at 10.0% FPR	0.151±0.133 vs 0.054±0.063, **p< 0.001**<br>AUC 0.783**, p< 0.001**, sen 96.97%, spe 51.09%,<br>cut off >0.0270, 33.33% at 10.0% FPR	0.152±0.180 vs 0.054±0.063, **p< 0.001**<br>AUC 0.724**, p< 0.001**, sen 83.05%, spe 52.17%,<br>cut off >0.0280, 30.51% at 10.0% FPR
miR-143-3p	0.068±0.114 vs 0.014±0.014, **p< 0.001**<br>AUC 0.863**, p< 0.001**, sen 100.00%, spe 59.78%,	0.036±0.025 vs 0.014±0.014, **p< 0.001**<br>AUC 0.839**, p< 0.001**, sen 93.94%, spe 63.04%,	0.049±0.053 vs 0.014±0.014, **p< 0.001**<br>AUC 0.811**, p< 0.001**, sen 67.80%, spe 83.70%,

	cut off >0.0114, 52.63% at 10.0% FPR	cut off >0.0122, 42.42% at 10.0% FPR	cut off >0.0233, 49.15% at 10.0% FPR
miR-145-5p	0.150±0.198 vs 0.063±0.039, p= 0.193<br>-	0.106±0.069 vs 0.063±0.039, **p= 0.014**<br>AUC 0.687, **p< 0.001**, sen 75.76%, spe 53.26%,<br>cut off >0.0601, 33.33% at 10.0% FPR	0.118±0.113 vs 0.063±0.039, **p= 0.011**<br>AUC 0.648, **p= 0.002**, sen 50.85%, spe 76.09%,<br>cut off >0.0826, 33.90% at 10.0% FPR
miR-146a-5p	1.766±1.555 vs 1.022±1.054, **p= 0.016**<br>AUC 0.742, **p< 0.001**, sen 84.21%, spe 64.13%,<br>cut off >0.9569, 15.79% at 10.0% FPR	1.922±1.291 vs 1.022±1.054, **p< 0.001**<br>AUC 0.761, **p< 0.001**, sen 87.88%, spe 56.52%,<br>cut off >0.7917, 42.42% at 10.0% FPR	2.080±1.810 vs 1.022±1.054, **p< 0.001**<br>AUC 0.761, **p< 0.001**, sen 66.10%, spe 77.17%,<br>cut off >1.3766, 38.98% at 10.0% FPR
miR-155-5p	1.980±1.225 vs 1.779±1.590, p= 1.000<br>-	1.835±1.545 vs 1.779±1.590, p= 1.000<br>-	1.877±1.637 vs 1.779±1.590, p= 1.000<br>-
miR-181a-5p	0.489±0.777 vs 0.216±0.312, **p= 0.003**<br>AUC 0.773, **p< 0.001**, sen 78.95%, spe 70.65%,<br>cut off >0.2226, 36.84% at 10.0% FPR	0.427±0.238 vs 0.216±0.312, **p< 0.001**<br>AUC 0.812, **p< 0.001**, sen 75.76%, spe 78.26%,<br>cut off >0.2678, 45.45% at 10.0% FPR	0.399±0.317 vs 0.216±0.312, **p< 0.001**<br>AUC 0.727, **p< 0.001**, sen 54.24%, spe 82.61%,<br>cut off >0.2972, 38.98% at 10.0% FPR
miR-195-5p	0.186±0.170 vs 0.069±0.109, **p< 0.001**<br>AUC 0.789, **p< 0.001**, sen 84.21%, spe 71.74%,<br>cut off >0.0586, 31.58% at 10.0% FPR	0.265±0.382 vs 0.069±0.109, **p< 0.001**<br>AUC 0.823, **p< 0.001**, sen 81.82%, spe 70.65%,<br>cut off >0.0553, 48.48% at 10.0% FPR	0.343±0.798 vs 0.069±0.109, **p< 0.001**<br>AUC 0.800, **p< 0.001**, sen 83.05%, spe 66.30%,<br>cut off >0.0483, 38.98% at 10.0% FPR
miR-199a-5p	0.075±0.043 vs 0.048±0.090, **p= 0.002**<br>AUC 0.780, **p< 0.001**, sen 89.47%, spe 64.13%,<br>cut off >0.0303, 21.05% at 10.0% FPR	0.100±0.123 vs 0.048±0.090, **p< 0.001**<br>AUC 0.757, **p< 0.001**, sen 72.73%, spe 78.26%,<br>cut off >0.0491, 15.15% at 10.0% FPR	0.111±0.139 vs 0.048±0.090, **p< 0.001**<br>AUC 0.784, **p< 0.001**, sen 79.66%, spe 69.57%,<br>cut off >0.0331, 27.12% at 10.0% FPR

miR-210-3p	0.098±0.114 vs 0.098±0.072, p= 1.000<br>-	0.091±0.064 vs 0.098±0.072, p= 1.000<br>-	0.084±0.066 vs 0.098±0.072, p= 0.912<br>-
miR-221-3p	0.662±0.594 vs 0.302±0.263, **p= 0.002**<br>AUC 0.768**, p< 0.001**, sen 100.00%, spe 46.74%,<br>cut off >0.1961, 31.58% at 10.0% FPR	0.683±0.554 vs 0.302±0.263, **p< 0.001**<br>AUC 0.778**, p< 0.001**, sen 90.91%, spe 55.43%,<br>cut off >0.2666, 33.33% at 10.0% FPR	0.681±0.562 vs 0.302±0.263, **p< 0.001**<br>AUC 0.786**, p< 0.001**, sen 74.58%, spe 70.65%,<br>cut off >0.3469, 37.29% at 10.0% FPR
miR-342-3p	3.472±5.160 vs 3.225±2.650, p= 1.000<br>-	3.534±1.870 vs 3.225±2.650, p= 0.745<br>-	3.280±2.084 vs 3.225±2.650, p= 1.000<br>-
miR-499a-5p	1.090±2.834 vs 0.175±0.339, **p< 0.001**<br>AUC 0.827**, p< 0.001**, sen 73.68%, spe 82.61%,<br>cut off >0.2280, 47.37% at 10.0% FPR	0.582±0.629 vs 0.175±0.339, **p< 0.001**<br>AUC 0.819**, p< 0.001**, sen 81.82%, spe 71.74%,<br>cut off >0.1188, 48.48% at 10.0% FPR	0.733±0.418 vs 1.229±0.339, **p< 0.001**<br>AUC 0.835**, p< 0.001**, sen 81.36%, spe 72.83%,<br>cut off >0.1271, 47.46% at 10.0% FPR
miR-574-3p	0.151±0.176 vs 0.075±0.051, **p= 0.021**<br>AUC 0.712**, p= 0.001**, sen 68.42%, spe 70.65%,<br>cut off >0.0832, 21.05% at 10.0% FPR	0.129±0.072 vs 0.075±0.051, **p< 0.001**<br>AUC 0.765**, p< 0.001**, sen 84.85%, spe 63.04%,<br>cut off >0.0713, 27.27% at 10.0% FPR	0.152±0.144 vs 0.075±0.051, **p< 0.001**<br>AUC 0.740**, p< 0.001**, sen 72.88%, spe 63.04%,<br>cut off >0.0713, 30.51% at 10.0% FPR

MicroRNA gene expression is compared between individual groups using Kruskal-Wallis test. ROC curve analysis shows the potential of individual microRNAs to differentiate between children descending from normal term pregnancies and those descending from preterm pregnancies with regard to gestational age at delivery. Statistically significant results are marked in bold. Mean ± SD values of relative fold gene expression of samples ($2^{-\Delta\Delta Ct}$) are presented. NP, normal pregnancies; ROC, receiver operating characteristic; AUC, area under the ROC curve; sen, sensitivity; spe, specificity; FPR, false positive rate.
